# An ontology for immune epitopes: application to the design of a broad scope database of immune reactivities

**DOI:** 10.1186/1745-7580-1-2

**Published:** 2005-09-20

**Authors:** Muthuraman Sathiamurthy, Bjoern Peters, Huynh-Hoa Bui, John Sidney, John Mokili, Stephen S Wilson, Ward Fleri, Deborah L McGuinness, Philip E Bourne, Alessandro Sette

**Affiliations:** 1 La Jolla Institute of Allergy and Immunology, 3030 Bunker Hill Street, Suite 326, San Diego, California, 92109, USA; 2 Knowledge Systems, Artificial Intelligence Laboratory, Stanford University and McGuinness Associates, Stanford, CA 94305, USA; 3 San Diego Supercomputer Center, P.O. Box 85608, San Diego, California 92186-5608, USA

## Abstract

**Background:**

Epitopes can be defined as the molecular structures bound by specific receptors, which are recognized during immune responses. The Immune Epitope Database and Analysis Resource (IEDB) project will catalog and organize information regarding antibody and T cell epitopes from infectious pathogens, experimental antigens and self-antigens, with a priority on NIAID Category A-C pathogens () and emerging/re-emerging infectious diseases. Both intrinsic structural and phylogenetic features, as well as information relating to the interactions of the epitopes with the host's immune system will be catalogued.

**Description:**

To effectively represent and communicate the information related to immune epitopes, a formal ontology was developed. The semantics of the epitope domain and related concepts were captured as a hierarchy of classes, which represent the general and specialized relationships between the various concepts. A complete listing of classes and their properties can be found at .

**Conclusion:**

The IEDB's ontology is the first ontology specifically designed to capture both intrinsic chemical and biochemical information relating to immune epitopes with information relating to the interaction of these structures with molecules derived from the host immune system. We anticipate that the development of this type of ontology and associated databases will facilitate rigorous description of data related to immune epitopes, and might ultimately lead to completely new methods for describing and modeling immune responses.

## Background

An epitope can be defined as the molecular structure recognized by the products of immune responses. According to this definition, epitopes are the specific molecular entities engaged in binding to antibody molecules or specific T cell receptors. An extended definition also includes the specific molecules binding in the peptide binding sites of MHC receptors. We have previously described [1] the general design of the Immune Epitope Database and Analysis Resource (IEDB), a broad program recently initiated by National Institute of Allergy and Infectious Diseases (NIAID). The overall goal of the IEDB is to catalog and organize a large body of information regarding antibody and T cell epitopes from infectious pathogens and other sources [2]. Priority will be placed on NIAID Category A-C pathogens () and emerging/re-emerging infectious diseases. Epitopes of human and non-human primates, rodents, and other species for which detailed information is available will be included. It is envisioned that this new effort will catalyze the development of new methods to predict and model immune responses, will aid in the discovery and development of new vaccines and diagnostics, and will assist in basic immunological investigations.

The IEDB will catalog structural and phylogenetic information about epitopes, information about their capacity to bind to specific receptors (i.e. MHC, TCR, BCR, Antibodies), as well as the type of immune response observed following engagement of the receptors (RFP-NIH-NIAID-DAIT-03/31: ).

In broad terms, the database will contain two general categories of data and information associated with immune epitopes – intrinsic and extrinsic (context-dependent data). Intrinsic features of an epitope are those characteristics that can be unequivocally defined and are specified within the epitope sequence/structure itself. Examples of intrinsic features are the epitope's sequence, structural features, and binding interactions with other immune system molecules. To describe an immune response associated with a specific epitope, context information also needs to be taken into account. Contextual information includes, for example, the species of the host, the route and dose of immunization, the health status and genetic makeup of the host, and the presence of adjuvants. In this respect, the IEDB project transcends the strict boundaries of database development and reaches into a systems biology application, attempting for the first time to integrate structural information about epitopes with comprehensive details describing their complex interaction with the immune system of the host, be it an infected organism or a vaccine recipient [1-3].

For these reasons, it was apparent at the outset of the project that it was crucial to develop a rigorous conceptual framework to represent the knowledge related to the epitopes. Such a framework was key to sharing information and ideas among developers, scientists, and potential users, and to allowing the design of an effective logical structure of the database itself. Accordingly, we decided to develop a formal ontology. Over the years, the term "ontology" has been defined and utilized in many ways by the knowledge engineering community [4]. We will adopt the definition of "ontology" as "the explicit formal specifications of the terms in a domain and the relationships among them" [5]. According to Noy and McGuinness [6], "ontology defines a common vocabulary for researchers who need to share information in a domain and helps separate domain knowledge from operational knowledge". Thus, availability of a formal ontology is relevant in designing a database, in cataloging the information, in communicating the database structure to researchers, developers and users, and in integrating multiple database schema designs and applications.

Several existing databases catalog epitope related data. We gratefully acknowledge that we have been able use these previous experiences in the design and implementation of the IEDB. MHCPEP [7], SYFPEITHI [8], FIMM [9], HLA Ligand Database [10], HIV Immunology Database [11], JenPep [12], AntiJen [13], and MHCBN [14] are all publicly available epitope related databases. In general, these databases provide information relating to epitopes, but do not catalog in-depth information relating to their interactions with the host's immune system. It should also be noted that none of these databases has published a formal ontology, but all of them rely on informal or implicit ontologies. We have taken into account as much as possible these ontologies, inferring their structure by informal communications with database developers or perusal of the databases websites.

The ontology developed for IEDB and described herein complements two explicit ontologies that are presently available: the IMGT-Ontology and the Gene Ontology (GO). The IMGT-Ontology [15] was created for the international ImMunoGeneTics Database (IMGT), which is an integrated database specializing in antigen receptors (immunoglobulin and T Cell receptors) and MHC molecules of all vertebrate species. This is, to the best of our knowledge, the first ontology in the domain of immunogenetics and immunoinformatics. The GO project [16] provides structured, controlled vocabularies that cover several domains of molecular and cellular biology. GO provides an excellent framework for genes, gene products, and their sequences, but it does not address the specific epitope substructure of the gene products. The IMGT provides an excellent ontological framework for the immune receptors but lacks information relating to the epitopes themselves. Therefore it was necessary to expand the available ontologies and to create an ontology specifically designed to represent the information of immune receptor interaction with immune epitopes. Wherever possible, the IEDB ontology conforms to standard vocabularies for capturing values for certain fields. For capturing disease names, IEDB uses the International Classification for Diseases (ICD-10) [17]. The NCBI Taxonomy database nomenclature [18,19] is used to capture species and strain names, and HLA Allele names are consistent with the HLA nomenclature reports [20].

## Construction and Content

The IEDB is being developed as a web-accessible database using Oracle 10g and Enterprise Java (J2EE). Industry standard software design has been followed and it is expected that IEDB will be available for public users by the end of 2005.

Protégé  was used to design and document the IEDB ontology. Protégé is a free, open source ontology editor and knowledge-base framework, written in Java. It provides an environment for creating ontologies and the terms used in those ontologies. Protégé supports class, slot, and instance creation, allowing users to specify relationships between appropriate entities. Two features that IEDB ontology effort used extensively were Protégé's support for creating ontology terms and for viewing the term hierarchies and the definitions. The support for a central repository on ontologies, along with browsing support, is key in reviewing and reusing ontologies.

While there are several open source tools available [21] for developing ontologies, we selected Protégé because of its extensibility to a variety of plug-ins that are readily available for integration. It also has the ability to export to different formats including the Ontology Web Language (OWL) (), which allows interoperability with other ontologies.

**Figure 1:**
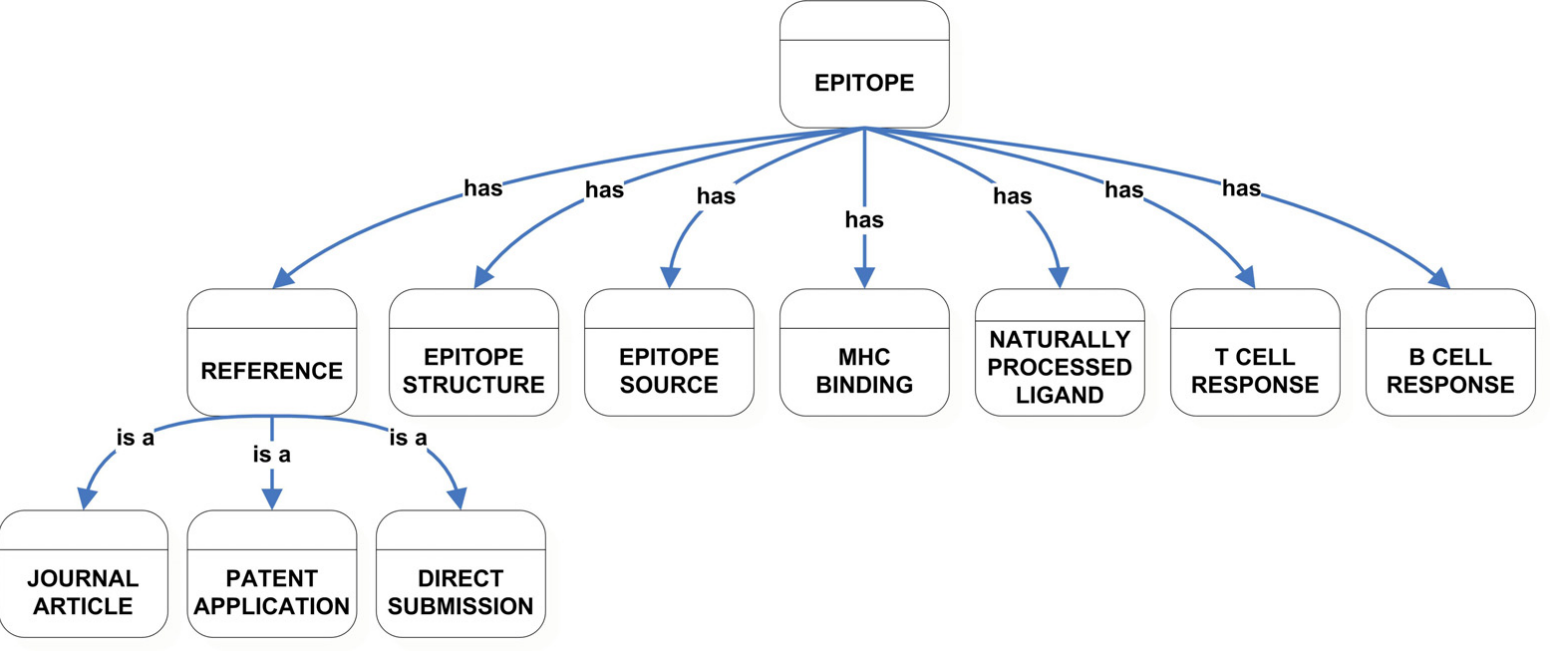
Overview of IEDB Class Hierarchy.

We have previously described some of the general concepts relating to the IEDB design [1,2]. More information relating to various aspects of the project can be accessed at . Herein, we report a detailed description of the novel aspects of the IEDB ontology. In designing our application architecture, we have followed the common system engineering practice of first determining the scope and nature of the data involved. A first essential step is to understand the semantics of the domain and to capture that knowledge in an agreed-upon format. Arranging the domain concepts in a taxonomy is one of the initial organizing steps in the ontology design process. The class hierarchy represents the generalization and specialization relationships between the various classes of objects in a domain [6]. Briefly, classes describe concepts in the domain. Subclasses represent concepts (classes) that are more specific than the superclass and these subclasses can have their own unique properties. Slots represent properties of the classes. For example, in Figure 1, we see that there is a class named Reference and three more specific subclasses of Reference: Journal Article, Patent Application, and Direct Submission. Figure 1 also shows that the class Epitope has a number of properties (slots) associated with it such as "has Epitope Structure" and "has Epitope Source".

### The IEDB Ontology: Reference, Epitope Structure, Epitope Source, and Assay Information classes

Our approach for creating the class hierarchy was a top-down development process where we defined each class in a domain and then identified its properties before building the hierarchy. The main classes identified for IEDB are Reference, Epitope Structure, Epitope Source, MHC Binding, Naturally Processed Ligand, T Cell Response, and B Cell Response (Figure 1). The Epitope class is the main class that encompasses all the individual concepts that were identified. The individual concepts are related to other classes. The primary relationships use the sub-class relationship or use a property (shown in the figures by the arcs labeled "has") that has a restriction on the type of the value that may fill that slot.

**Figure 2:**
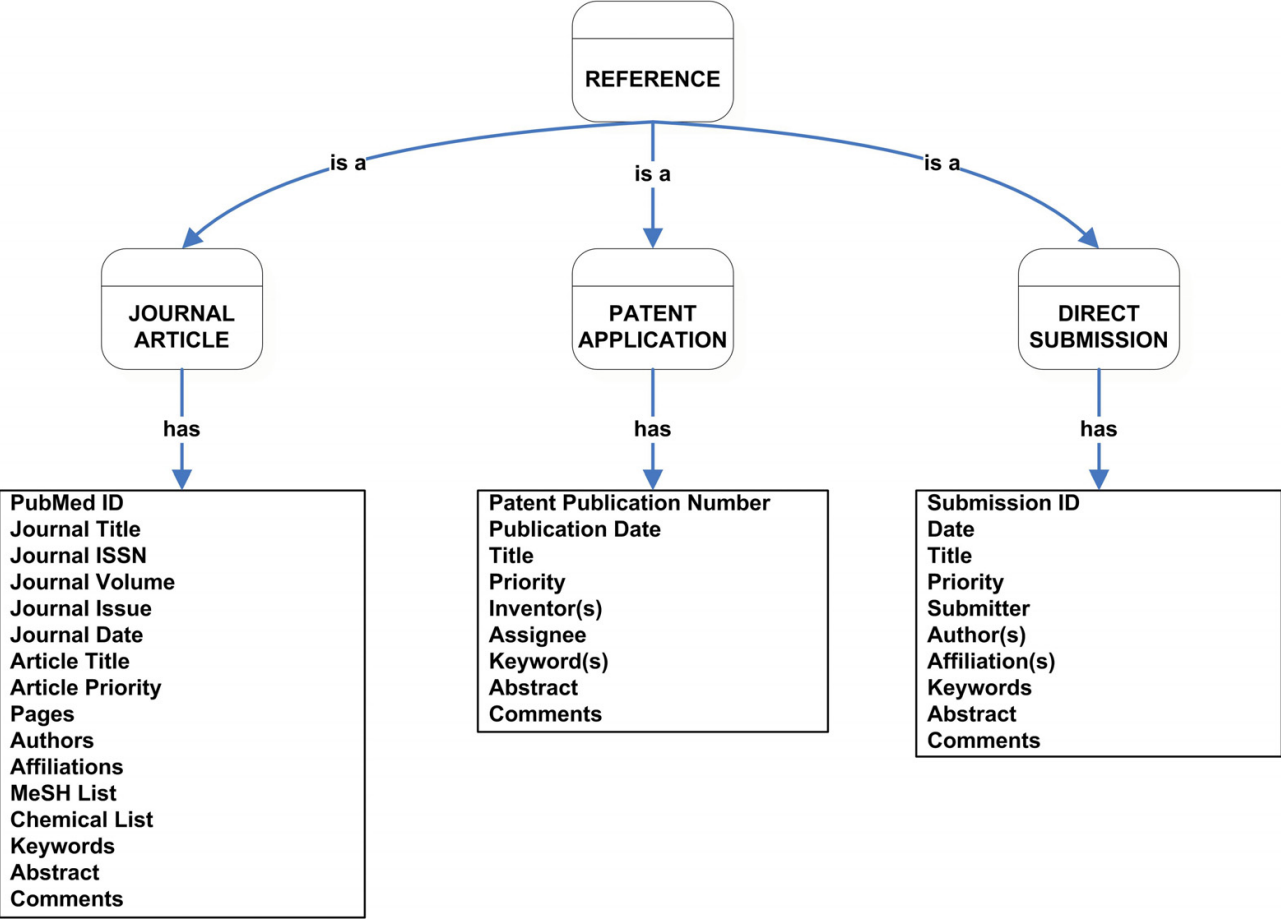
Detailed classification of Reference class showing its subclasses and slots.

"Reference" is the class encompassing information related to the data source from which an epitope and its related information are extracted into the IEDB. We have identified three broad subclasses of References that describe where epitope information will be obtained. They are Journal Article, Patent Application, and Direct Submission. The complete listing of slots (fields) encompassed by the Journal Article, Patent Application, and Submission classes are provided in Figure 2. The Journal Article class refers to manuscripts published in peer-reviewed journals. The Patent Application class captures all the reference fields for a patent application that contain epitope information. The Submission class captures information about sources that contribute data to the IEDB directly. Data deposited by the Large Scale Antibody and T Cell Epitope Discovery contracts [3] and those transferred from other websites fall into this class.

**Figure 3:**
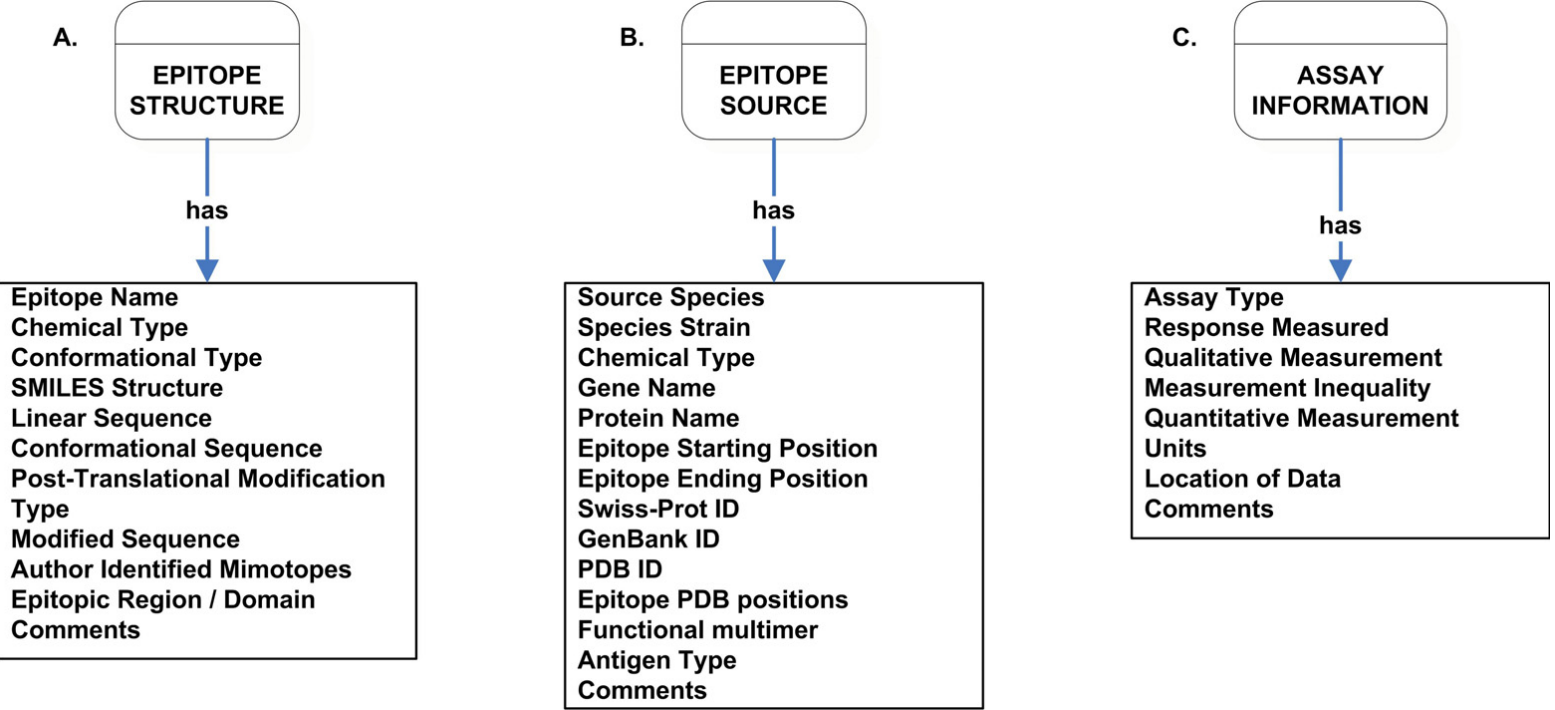
Detailed listing of properties of Epitope Structure (A), Epitope Source (B), and Assay Information (C) class.

The Epitope Structure and Epitope Source classes capture intrinsic features of an epitope. The Epitope Structure class captures the physical and chemical features of an epitope. Virtually any molecular structure may provoke an immune response, such as proteins, carbohydrates, DNA, and lipids. In the Epitope Structure class, structural information relating to linear sequences and 2-D structures of the epitope, if available, are catalogued. The Epitope Source class captures the phylogenetic source of an epitope, including species of origin, gene name, protein name, and links to other databases for more detailed information about proteins and genes. Figures 3A and 3B show the listings of properties (slots/fields) encompassing the Epitope Structure and Epitope Source classes.

The experimental data and information about specific experiments and the methodology utilized are captured in the Assay Information class. The name of the assay used, the type of response measured in the assay, and the readout of the assay are examples of information captured in the Assay Information class. This important class is used as a superclass of several other classes (and thus its properties are inherited by those classes). A complete listing the properties (slots/fields) in the Assay Information class is shown in Figure 3C.

### Immunization, Antigen, and Antigen Presenting Cell classes

**Figure 4:**
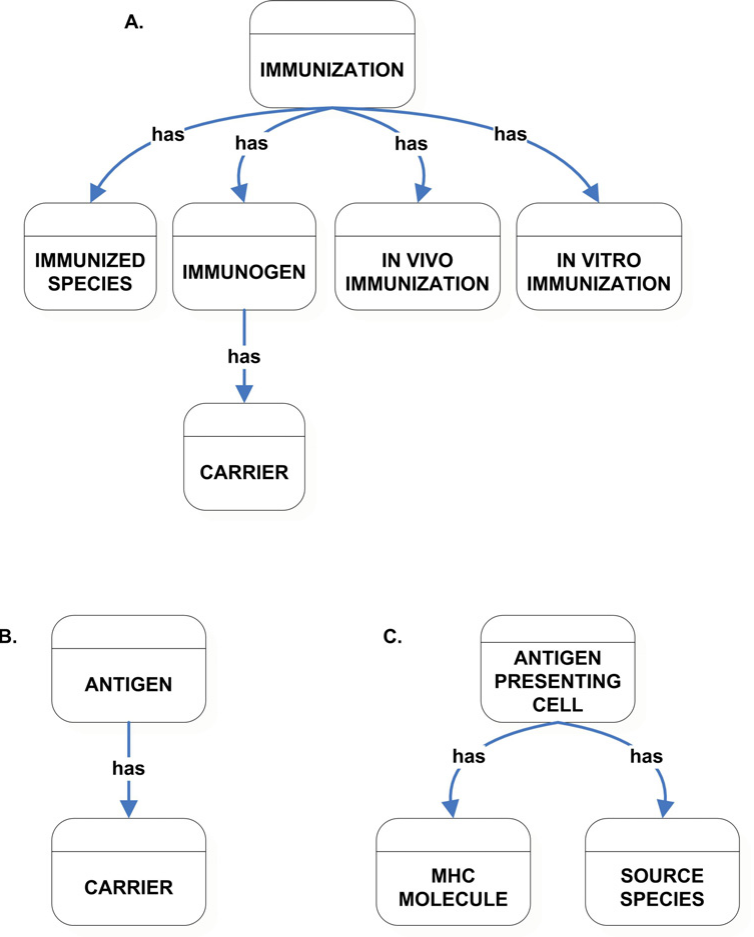
High-level classification of Immunization (A), Antigen (B), and Antigen Presenting Cells (C) class.

As with Assay Information, the classes Immunization, Antigen, and Antigen Presenting Cell are used in multiple other class descriptions. Features relating to the induction of the immune response are captured in the Immunization class (Figure 4A). It has relationships to other classes like Immunized Species, Immunogen, In vivo Immunization, and In vitro Immunization. Immunized Species contains information relating to the host that is being immunized. The Immunogen class describes the molecules that induce the immune response and an associated carrier molecule, if present. Features relating to how the immunogen was introduced to the immunized species are captured under the In vivo and In vitro Immunization classes.

Similarly, antigens are defined as the whole molecules that react with the products of an immune response (as opposed to the epitopes which are the specific structures, contained within the antigen that engages the immune receptor). Information relating to the antigen and any associated carrier molecule is captured in the Antigen class (Figure 4B). During immune responses, antigen-presenting cells process antigens and present peptide epitopes complexed with MHC molecules. This information is captured in the Antigen Presenting Cells class, which has a relationship to the MHC Molecules and the Source Species classes (Figure 4C). The Source Species class describes the species information from which the antigen presenting cells are derived.

### The MHC Binding, Naturally Processed Ligand, T Cell Response, and B Cell Response classes

**Figure 5:**
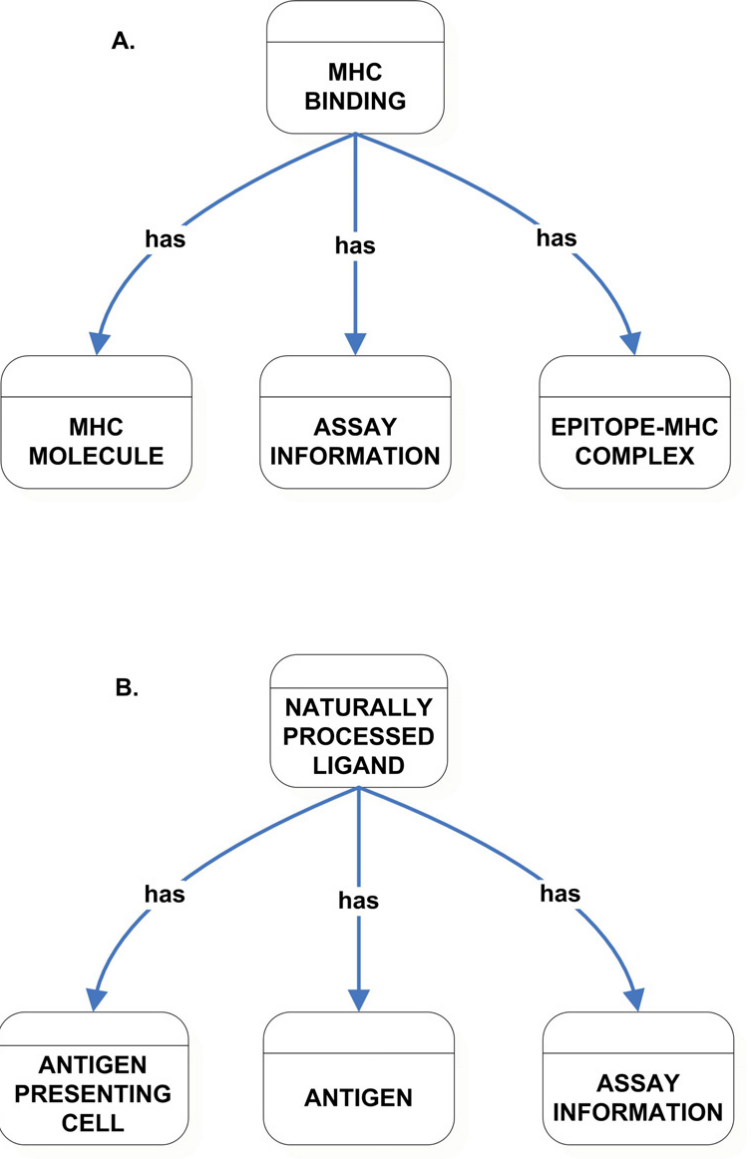
High-level classification of MHC Binding (A) and Naturally Processed Ligand (B) class.

The MHC Binding class captures the details relating to the interaction of the epitope with specific MHC molecules and information relating to the MHC molecule along with any available Epitope-MHC complex structure details. This class also has a slot that is restricted to be an instance of the Assay Information class (Figure 5A).

Extrinsic features of an epitope are captured by Naturally Processed Ligand, T Cell Response, and B Cell Response classes. Extrinsic features are context-dependent attributes, being dependent upon specific experimental conditions. The Naturally Processed Ligand class captures data related to epitopes that are naturally processed and presented on the cell surface. This class has properties that are instances of classes including Antigen Presenting Cell, Antigen, and Assay Information (Figure 5B).

The Naturally Processed Ligand class differs from the MHC Binding class in that information related to the antigen that was processed and the cell types in which the processing occurred is represented. MHC Binding class captures data relating to in vitro MHC binding assays, which assess the epitope's binding capacity to the MHC molecule. Hence the MHC Binding class does requires neither the Antigen class not the Antigen Presenting Cells class. In general, naturally processed ligands are assessed in the absence of a T cell response, for example, identified by direct elution from MHC molecules extracted from infected cells or antigen presenting cells. Thus, the Immunization class is not used as a value restriction by the Naturally Processed Ligand class.

**Figure 6:**
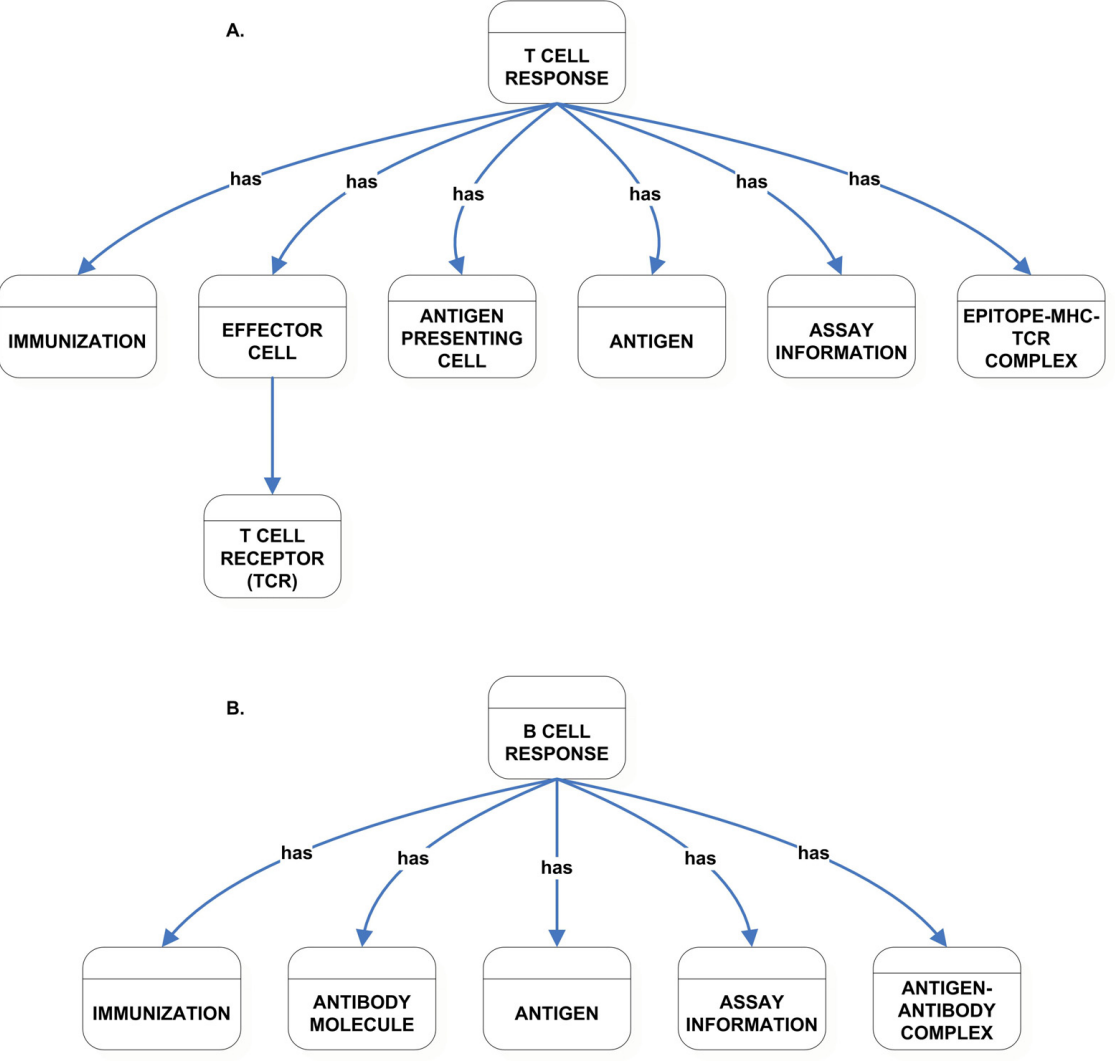
High-level classification of T Cell Response (A) and B Cell Response (B) class.

The T Cell Response class captures all of the T cell mediated immunity-related information (Figure 6A). It has properties that are of type: Immunization, Effector Cells, Antigen Presenting Cell, Antigen, Assay Information, and Epitope-MHC-TCR Complex. The Effector Cell class describes the cells that are elicited upon immunization and that acquire measurable functions as a result. The B Cell Response class describes antibody responses that are related to the epitope (Figure 6B). This class has properties that are of type: Immunization, Antibody Molecule, Antigen, Assay Information, and Antigen-Antibody Complex. Because B cell responses do not require MHC binding and antigen presenting cells, the respective classes related to MHC Molecule and Antigen Presenting Cells are not used as restrictions on properties of the B Cell Response class.

### Classes capturing 3D structures

There are three classes that capture information about the 3D structure of complexes: Epitope-MHC Complex, Epitope-MHC-TCR Complex, and Antigen-Antibody Complex. The Epitope-MHC Complex, Epitope-MHC-TCR Complex, and Antigen-Antibody Complex classes are used as restrictions on properties of the MHC Binding, T Cell Response, and B Cell Response classes respectively (Figures 5A, 6A, and 6B). These Complex classes capture the Protein Data Bank (PDB) Identifier, which provides detailed information about 3D structures. The Protein Data Bank [22,23] contains approximately 1600 3D structures that are of immunological interest. Other information that is not available in PDB, such as the atom pairs that are involved in the interactions between molecules, the specific residues, the contact area of the molecules, and allosteric effect, is also captured here.

### IEDB Class Hierarchy and Data Dictionary

Each class has numerous slots that capture detailed information associated with epitopes. As mentioned above, a complete list of all the classes, their properties, and relationship, can be found at . One of the files provided as supplementary material contains two examples of how two literature references [24,25] containing epitope information are extracted into the IEDB ontology (additional file 1). Along with the class hierarchy, the IEDB's data dictionary (additional file 2) provides more detailed information about the fields that are defined for the IEDB. The data dictionary contains a textual overview description and a listing of fields that are required to be completed for IEDB entries. The data dictionary also allows database users to provide comments and suggestions to IEDB team to enhance the formal ontology.

## Utility and Discussion

The IEDB will be a comprehensive resource pertaining to epitopes of the immune system. By extensively curating both intrinsic and extrinsic features associated with epitopes, the IEDB is expected to provide substantially greater detail about specific epitopes than any other databases presently available. The IEDB will be populated with data derived from three main sources, namely the peer-reviewed literature, patent applications, and direct submission. Epitope data published in the literature and patent applications are curated manually by the IEDB's curation team. Data from already existing epitope databases, whose authors have agreed to share their data, will also be imported into the IEDB. Apart from these, a main data source will be the direct submission of data from the Large-scale Antibody and T Cell Epitope Discovery programs [3] that are funded by NIAID. Presently, fourteen contracts have been awarded under this program, and all of them will submit their data to the IEDB. Direct antibody and T cell epitope submissions will also be sought from the broader research community, with an emphasis on antibody epitopes to NIAID Category A-C pathogens. Because of the large scale of the IEDB project, a formal ontology is critical to ensure consistency in the representation of data.

Communication between database developers, researchers, analysis tool developers, and team members is crucial, and can be performed in harmony only when a common vocabulary is established. An ontology, which is an explicit formal specification of the terms in the domain and relationships among them, is an effective way to share the knowledge contained in that domain. Accordingly, since the IEDB's domain is epitope-related data, we have created an ontology that captures detailed conceptual structure related to these data.

The development of this ontology has relevance for the expansion and modification of the epitope knowledge base. Our ontology design defines individual concepts as separate classes and then defined relationships between these classes and other objects in the domain. These classes serve to restrict the values that will describe properties of objects in the database. For example, the species is a separate concept defined in its own class. Depending on the context, this can refer to an immunized species or the species from which antigen presenting cells are derived. Similarly MHC Molecules is defined as a separate class, and it is used as a value restriction by concepts like MHC Binding and Antigen Presenting Cells. Defining concepts as separate classes and using them to restrict the values of properties in other classes facilitates the expansion and modification of our ontology. Adding properties (slots) to concepts is a task easily accomplished when there are well-defined class descriptions that may serve as value restrictions on the properties, and providing that these class descriptions are general enough to apply in all instances. We have ensured in our design that each concept is atomic and that it can be re-used by various classes.

The development of a formal ontology is valuable to database users and in particular to scientists contributing data to, and downloading data from, the IEDB. We anticipate that the availability of a formal ontology will ensure that a common language and shared understanding of concepts will inspire this type of communication, thus ensuring maximum efficiency and accuracy. The formal ontology developed will most likely require refinement and fine tuning when users provide suggestions and new technologies for performing experiments are discovered. The IEDB website will provide mechanisms for the users to provide suggestions and participate in the enhancement of the ontology. The IEDB Data Dictionary has a separate column for the users to provide comments on specific data fields. The IEDB website will also host web forms that will guide users to conform to the ontology definitions when submitting data. Apart from the web forms, an XML schema definition (XSD) will be available on the website for users to inspect and use in their data submission. Users will also be able to download epitope records from the website.

In the process of developing new ontologies, it is good practice to leverage existing community standards. In our initial analysis, we confirmed that there were no explicit ontologies that efficiently captured epitope details as per the scope of the IEDB program. As mentioned above, we have utilized, as much as possible, inferred ontologies from existing epitope databases. Among the ontologies that we analyzed, IMGT-Ontology and Gene Ontology were the only two formal ontologies that were related to the epitope domain. The IMGT-Ontology was designed for the ImMunoGeneTics database. IMGT is an integrated database specializing in antigen receptors (immunoglobulins and T-cell receptors) and the major histocompatibility complex of all vertebrate species. The ontology developed for this database has specific immunological content, describing the classification and specification of terms needed for immunogenetics. The IEDB does conform to IMGT's standards about receptors and MHC molecule chains in the sense that all the chain names follow IMGT's controlled vocabulary.

GO provides structured controlled vocabularies for genes, gene products, and sequences annotated for many organisms. The IEDB complements GO in terms of epitopes of immunological interest since GO is incomplete in this area. Antigens, which are primary sources of epitopes, are annotated in GO. Thus, in essence, the IEDB could be utilized to provide an extension of GO for antigens that contain epitope-related information.

## Conclusion

Perhaps the most important element in the development of the IEDB ontology is that, to the best of our knowledge, this represents the first immunological ontology specifically designed to capture both intrinsic biochemical and extrinsic context dependent information. In this respect, it is similar in spirit, but different in approach, from other knowledge resources relating to systems biology. We anticipate that the development of this type of ontology and associated databases might lead to completely new methods for describing and modeling immune responses. Accordingly, this new program might represent a novel tool to assist in the design, testing, and development of new ways to combat infectious diseases and other immune related pathologies such as cancer and autoimmune diseases.

## Availability

A complete listing of IEDB's class hierarchy and its properties is available at 

## List of Abbreviations Used

IEDB – Immune Epitope Database and Analysis Resource

MHC – Major Histocompatibility Complex

TCR – T Cell Receptor

BCR – B Cell Receptor

IMGT – Immunogenetics Database

GO – Gene Ontology

J2EE – Java 2 Enterprise Edition

OWL – Ontology Web Language

PDB – Protein Data Bank

NIAID – National Institute of Allergy and Infectious Diseases

XML – Extensible Markup Language

XSD – XML Schema Definition

## Authors' contributions

MS generated the formal ontology using Protégé. MS, BP, HB, JS, and AS designed the initial ontology. SW, JM, WF, DM, PB provided critical insight in enhancing the initial design and creating the formal ontology. All authors participated in the preparation of the manuscript.

## Supplementary Material

Additional File 1 Sample Curation. This file contains a table that shows how epitope and its related information were extracted from a literature reference and mapped into the IEDB ontology. Click here for file

Additional File 2 IEDB Data Dictionary v12-5. The Data Dictionary contains the textual overview description and a listing of fields that are captured for IEDB. Click here for file
