# Supplementary material for: An ontology for immune epitopes: application to the design of a broad scope database of immune reactivities
Source: Immunome Res. 2005 Sep 20;1:2. doi: 10.1186/1745-7580-1-2 (PMC1287064; doi:10.1186/1745-7580-1-2)
Supplement: Additional File 1 — Sample Curation. This file contains a table that shows how epitope and its related information were extracted from a literature reference and mapped into the IEDB ontology. [file 1745-7580-1-2-S1.pdf]

Sample Curation of Epitope Data into IEDB Ontology

This report shows how epitopes and their related information were extracted from two literature references (PubMed ID: 12668642 and 12862314) into the Immune Epitope Database and Analysis Resource (IEDB) Ontology. Only the fields for which data were extracted are displayed in this report. A complete listing of IEDB fields along with explanation of their meaning can be found at http://www.immuneepitope.org/ontology/index.html.

Curation of Literature Reference (PubMed ID : 12668642)

REFERENCE – ARTICLE	
PUBMED ID	12668642
Title	Quantitation of CD8+ T cell responses to newly identified HLA-A*0201-restricted T cell epitopes conserved among vaccinia and variola (smallpox) viruses.
Author	Terajima M, Cruz J, Raines G, Kilpatrick ED, Kennedy JS, Rothman AL, Ennis FA
Pub Date	2003 Apr

EPITOPE 1 of 2	
**EPITOPE STRUCTURE**	
Epitope Name	74A
Chemical Type	Peptide / Protein
Continuous Epitope	Continuous
Linear Sequence	CLTEYILWV
Author Identified Mimotope	No
**EPITOPE SOURCE**	
Epitopic Region / Domain	No
Source Species	Vaccinia Virus
Strain	Ankara
Chemical Type of Source Antigen	Peptide / Protein
Protein Name	21.7 K protein
GenBank ID	2772819
Epitope Starting Position	79
Epitope Ending Position	87
**MHC BINDING**	**No data for Epitope 1**
**IMMUNE RESPONSE - T Cell Response Data 1 for EPITOPE 1**	
**IMMUNIZATION**	
**Immunized Species**	
Species	Homo Sapiens
Disease Name	Healthy
Immunization category	Administration
**Immunogen**	
Immunogen Type	Source Species
Name	Dryvax Vaccine
Source Species	Vaccinia Virus
Strain	New York City Board of Health
**In vivo Immunization**	
Formulation	Virus Suspension
Administration Route	Scarification
Dose Schedule	Donors received primary immunization
**In vitro Immunization / Restimulation**	
Responder Cells	PBMC
Stimulator Cells	PBMC
Restimulation Comments	CTL lines were established from a single donor
**ASSAY**	
**Effector Cells**	

Effector Cells	CTL
Origin	Cell line / Clone
**Antigen Presentation**	
MHC Allele	HLA A*0201
**Species of Antigen Presenting Cells**	
APC - Autologous or Syngeneic?	No
**Antigen Presenting Cells**	
Antigen Presenting Cells	C1R cells
Origin	Cell line / Clone
**Antigen**	
Antigen Type	Epitope
**Assay Information**	
Assay Type	51 Chromium Release
Type of Response Measured	Killing
Qualitative Measurement	Positive
Units	% SL
Number of Subjects Tested	1
Number of Subjects Responded	1
LOCATION OF DATA	Figure 1
**IMMUNE RESPONSE - T Cell Response Data 2 for EPITOPE 1**	
**IMMUNIZATION**	
**Immunized Species**	
Species	Homo Sapiens
Disease Name	Healthy
Immunization category	Administration
**Immunogen**	
Immunogen Type	Source Species
Name	Dryvax Vaccine
Source Species	Vaccinia Virus
Strain	New York City Board of Health
**In vivo Immunization**	
Formulation	Virus Suspension
Administration Route	Scarification
Dose Schedule	Donors received primary immunization
**ASSAY**	
**Effector Cells**	
Effector Cells	CD8+ T Cells
Origin	Ex vivo
**Antigen Presentation**	
MHC Allele	HLA A*0201
**Antigen**	
Antigen Type	Epitope
**Assay Information**	
Assay Type	FACS / MHC Tetramer Staining
Type of Response Measured	TCR Binding
Qualitative Measurement	Positive
Units	% Population
Number of Subjects Tested	3
Number of Subjects Responded	3
LOCATION OF DATA	Figure 2, 3
COMMENTS ON ASSAY	In all three donors, the frequency of vaccinia specific CD8+ T cells peaked 2 weeks after primary immunization and then declined, but was still detectable 1 to 3 years after primary immunization.

IMMUNE RESPONSE - T Cell Response Data 3 for EPITOPE 1	
**IMMUNIZATION**	
**Immunized Species**	
Species	Homo Sapiens
Disease Name	Healthy
Immunization category	Administration
**Immunogen**	
Immunogen Type	Source Species
Name	Dryvax Vaccine
Source Species	Vaccinia Virus
Strain	New York City Board of Health
**In vivo Immunization**	
Formulation	Virus Suspension
Administration Route	Scarification
Dose Schedule	Donors received primary immunization
**ASSAY**	
**Effector Cells**	
Effector Cells	T Cells
Origin	Ex vivo
**Antigen Presentation**	
MHC Allele	HLA A*0201
**Species of Antigen Presenting Cells**	
APC - Autologous or Syngeneic ?	Yes
**Antigen Presenting Cells**	
Antigen Presenting Cells	PBMC
Origin	Ex vivo
**Antigen**	
Antigen Type	Epitope
**Assay Information**	
Assay Type	ELISPOT
Type of Response Measured	Cytokine Release (IFN-g)
Qualitative Measurement	Positive
Units	SFC
Number of Subjects Tested	3
Number of Subjects Responded	3
LOCATION OF DATA	Figure 4
COMMENTS ON ASSAY	IFN-g production peaked two weeks after primary immunization. Response was also shown to Vaccinia virus NYCBH strain
**Peptide Elution Data**	**No data for Epitope 1**
**IMMUNE RESPONSE - B Cell**	**No data for Epitope 1**

EPITOPE 2 of 2	
**EPITOPE STRUCTURE**	
Epitope Name	165
Chemical Type	Peptide / Protein
Continuous Epitope	Continuous
Linear Sequence	KVDDTFYYV
Author Identified Mimotope	No
**EPITOPE SOURCE**	
Epitopic Region / Domain	No
Source Species	Vaccinia Virus
Strain	Ankara
Chemical Type of Source Antigen	Peptide / Protein

Protein Name	Host range protein
GenBank ID	56405257
Epitope Starting Position	74
Epitope Ending Position	82
**MHC BINDING**	**No data for Epitope 2**
**IMMUNE RESPONSE - T Cell Response Data 1 for EPITOPE 2**	
**IMMUNIZATION**	
**Immunized Species**	
Species	Homo Sapiens
Disease Name	Healthy
Immunization category	Administration
**Immunogen**	
Immunogen Type	Source Species
Name	Dryvax Vaccine
Source Species	Vaccinia Virus
Strain	New York City Board of Health
**In vivo Immunization**	
Formulation	Virus Suspension
Administration Route	Scarification
Dose Schedule	Donors received primary immunization
**In vitro Immunization / Restimulation**	
Responder Cells	PBMC
Stimulator Cells	PBMC
Restimulation Comments	CTL lines were established from a single donor
**ASSAY**	
**Effector Cells**	
Effector Cells	CTL
Origin	Cell line / Clone
**Antigen Presentation**	
MHC Allele	HLA A*0201
**Species of Antigen Presenting Cells**	
APC - Autologous or Syngeneic ?	No
**Antigen Presenting Cells**	
Antigen Presenting Cells	C1R cells
Origin	Cell line / Clone
**Antigen**	
Antigen Type	Epitope
**Assay Information**	
Assay Type	51 Chromium Release
Type of Response Measured	Killing
Qualitative Measurement	Positive
Units	% SL
Number of Subjects Tested	1
Number of Subjects Responded	1
LOCATION OF DATA	Figure 1
**IMMUNE RESPONSE - T Cell Response Data 2 for EPITOPE 2**	
**IMMUNIZATION**	
**Immunized Species**	
Species	Homo Sapiens
Disease Name	Healthy
Immunization category	Administration
**Immunogen**	
Immunogen Type	Source Species

Name	Dryvax Vaccine
Source Species	Vaccinia Virus
Strain	New York City Board of Health
**In vivo Immunization**	
Formulation	Virus Suspension
Administration Route	Scarification
Dose Schedule	Donors received primary immunization
**ASSAY**	
**Effector Cells**	
Effector Cells	CD8+ T Cells
Origin	Ex vivo
**Antigen Presentation**	
MHC Allele	HLA A*0201
**Antigen**	
Antigen Type	Epitope
**Assay Information**	
Assay Type	FACS / MHC Tetramer Staining
Type of Response Measured	TCR Binding
Qualitative Measurement	Positive
Units	% Population
Number of Subjects Tested	3
Number of Subjects Responded	3
LOCATION OF DATA	Figure 2, 3
COMMENTS ON ASSAY	In all three donors, the frequency of vaccinia specific CD8+ T cells peaked 2 weeks after primary immunization and then declined, but was still detectable 1 to 3 years after primary immunization.
**IMMUNE RESPONSE - T Cell Response Data 3 for EPITOPE 2**	
**IMMUNIZATION**	
**Immunized Species**	
Species	Homo Sapiens
Disease Name	Healthy
Immunization category	Administration
**Immunogen**	
Immunogen Type	Source Species
Name	Dryvax Vaccine
Source Species	Vaccinia Virus
Strain	New York City Board of Health
**In vivo Immunization**	
Formulation	Virus Suspension
Administration Route	Scarification
Dose Schedule	Donors received primary immunization
**ASSAY**	
**Effector Cells**	
Effector Cells	T Cells
Origin	Ex vivo
**Antigen Presentation**	
MHC Allele	HLA A*0201
**Species of Antigen Presenting Cells**	
APC - Autologous or Syngeneic ?	Yes
**Antigen Presenting Cells**	
Antigen Presenting Cells	PBMC
Origin	Ex vivo
**Antigen**	

Antigen Type	Epitope
**Assay Information**	
Assay Type	ELISPOT
Type of Response Measured	Cytokine Release (IFN-g)
Qualitative Measurement	Positive
Units	SFC
Number of Subjects Tested	3
Number of Subjects Responded	3
LOCATION OF DATA	Figure 4
COMMENTS ON ASSAY	IFN-g production peaked two weeks after primary immunization. Response was also shown to Vaccinia virus NYCBH strain
**Peptide Elution Data**	**No data for Epitope 2**
**IMMUNE RESPONSE - B Cell**	**No data for Epitope 2**

Curation of Literature Reference (PubMed ID : 12862314)

REFERENCE - ARTICLE	
PUBMED ID	12862314
Title	Identification of an epitope of SARS-coronavirus nucleocapsid protein.
Author	Lin Y, Shen X, Yang RF, Li YX, Ji YY, He YY, Shi MD, Lu W, Shi TL, Wang J, Wang HX, Jiang HL, Shen JH, Xie YH, Wang Y, Pei G, Shen BF, Wu JR, Sun B.
Pub Date	2003 Jun

EPITOPE 1 of 2	
**EPITOPE STRUCTURE**	
Epitope Name	N1
Chemical Type	Peptide / Protein
Continuous Epitope	Continuous
Linear Sequence	PTDSTDNNQNGGRNGARPKQRRPQ
Author Identified Mimotope	No
**EPITOPE SOURCE**	
Epitopic Region / Domain	Yes
Source Species	SARS Coronavirus
Chemical Type of Source Antigen	Peptide / Protein
Protein Name	Nucleocapsid protein
GenBank ID	30173007
Swiss-Prot ID	P59595
Epitope Starting Position	21
Epitope Ending Position	44
**MHC BINDING**	**No data for Epitope 1**
**IMMUNE RESPONSE - T Cell**	**No data for Epitope 1**
**NATURALLY PROCESSED CONTEXT**	**No data for Epitope 1**
**IMMUNE RESPONSE - B Cell Response Data 1 for EPITOPE 1**	
**IMMUNIZATION**	
**Immunized Species**	
Species	Oryctolagus cuniculus
Strain / Ethnicity	New Zealand
Disease Name	Healthy
Immunization category	Administration
**Immunogen**	
Immunogen Type	Epitope
**Carrier / Vector**	
Carrier Name	Bovine serum ablbumin
Source Species	Bos taurus
**In vivo Immunization**	
Formulation	Peptide emulsion supplemented with Mycobacterium tuberculosis
Adjuvant(s)	Freund's complete; Freund's incomplete (IFA);
Administration Route	Subcutaneous (s.c)

Dose Schedule	Rabbits were injected multiple times on the back. Three weeks later, booster injections were administered with Freund's incomplete adjuvant
**ASSAY**	
**Antibody**	
Antibody Name	anti-N-protein
Antibody Type	Polyclonal
Source Species	Oryctolagus cuniculus
Strain	New Zealand
Isotype	IgG
**Antigen**	
Antigen Type	Source Protein
Antigen Name	Nucleocapsid protein
Chemical Type	Peptide / Protein
Source Species	SARS coronavirus
GenBank ID	30173007
SWISS-PROT ID	P59595
**Assay Information**	
Materials Assayed	serum
Assay Type	Western Blot (Immunoblot)
Type of Response Measured	Antibody Screening
Qualitative Measurement	Positive
LOCATION OF DATA	Figure 2
**IMMUNE RESPONSE - B Cell Response Data 2 for EPITOPE 1**	
**IMMUNIZATION**	
**Immunized Species**	
Species	Oryctolagus cuniculus
Strain / Ethnicity	New Zealand
Disease Name	Healthy
Immunization category	Administration
**Immunogen**	
Immunogen Type	Epitope
**Carrier / Vector**	
Carrier Name	Bovine serum ablbumin
Source Species	Bos taurus
**In vivo Immunization**	
Formulation	Peptide emulsion supplemented with Mycobacterium tuberculosis
Adjuvant(s)	Freund's complete; Freund's incomplete (IFA);
Administration Route	Subcutaneous (s.c)
Dose Schedule	Rabbits were injected multiple times on the back. Three weeks later, booster injections were administered with Freund's incomplete adjuvant
**ASSAY**	
**Antibody**	
Antibody Name	anti-N-protein
Antibody Type	Polyclonal
Source Species	Oryctolagus cuniculus
Strain	New Zealand
Isotype	IgG
**Antigen**	
Antigen Type	Epitope
**Carrier / Vector**	
Carrier Name	Bovine serum albumin

Source Species	Bos taurus
**Assay Information**	
Materials Assayed	serum
Assay Type	Western Blot (Immunoblot)
Type of Response Measured	Antibody Screening
Qualitative Measurement	Positive
LOCATION OF DATA	Table 1
COMMENTS ON ASSAY	BSA preabsorption and BSA antigen were conducted as controls with negative responses.
**IMMUNE RESPONSE - B Cell Response Data 3 for EPITOPE 1**	
**IMMUNIZATION**	
**Immunized Species**	
Species	Homo sapiens
Disease Name	SARS
Disease Stage	Unknown
Immunization category	Natural Infection or Exposure
**Immunogen**	
Immunogen Type	Source Species
Source Species	SARS coronavirus
**ASSAY**	
**Antibody**	
Antibody Name	anti-N-protein
Antibody Type	Polyclonal
Source Species	Homo sapiens
Isotype	IgG
**Antigen**	
Antigen Type	Epitope
**Carrier / Vector**	
Carrier Name	Bovine serum albumin
Source Species	Bos taurus
**Assay Information**	
Materials Assayed	serum
Assay Type	Western Blot (Immunoblot)
Type of Response Measured	Antibody Screening
Qualitative Measurement	Positive
Number of Subjects Tested	9
Number of Subjects Responded	3
LOCATION OF DATA	Table 2

**EPITOPE 2 of 2**	
**EPITOPE STRUCTURE**	
Epitope Name	N2
Chemical Type	Peptide / Protein
Continuous Epitope	Continuous
Linear Sequence	GALNTPKDHIGTRNPNNNAATVL
Author Identified Mimotope	No
**EPITOPE SOURCE**	
Epitopic Region / Domain	Yes
Source Species	SARS Coronavirus
Chemical Type of Source Antigen	Peptide / Protein
Protein Name	Nucleocapsid protein
GenBank ID	30173007

Swiss-Prot ID	P59595
Epitope Starting Position	138
Epitope Ending Position	44
**MHC BINDING**	**No data for Epitope 2**
**IMMUNE RESPONSE - T Cell**	**No data for Epitope 2**
**NATURALLY PROCESSED CONTEXT**	**No data for Epitope 2**
**IMMUNE RESPONSE - B Cell Response Data 1 for EPITOPE 2**	
**IMMUNIZATION**	
**Immunized Species**	
Species	Oryctolagus cuniculus
Strain / Ethnicity	New Zealand
Disease Name	Healthy
Immunization category	Administration
**Immunogen**	
Immunogen Type	Epitope
**Carrier / Vector**	
Carrier Name	Bovine serum ablbumin
Source Species	Bos taurus
**In vivo Immunization**	
Formulation	Peptide emulsion supplemented with Mycobacterium tuberculosis
Adjuvant(s)	Freund's complete; Freund's incomplete (IFA);
Administration Route	Subcutaneous (s.c)
Dose Schedule	Rabbits were injected multiple times on the back. Three weeks later, booster injections were administered with Freund's incomplete adjuvant
**ASSAY**	
**Antibody**	
Antibody Name	anti-N-protein
Antibody Type	Polyclonal
Source Species	Oryctolagus cuniculus
Strain	New Zealand
Isotype	IgG
**Antigen**	
Antigen Type	Source Protein
Antigen Name	Nucleocapsid protein
Chemical Type	Peptide / Protein
Source Species	SARS coronavirus
GenBank ID	30173007
SWISS-PROT ID	P59595
**Assay Information**	
Materials Assayed	serum
Assay Type	Western Blot (Immunoblot)
Type of Response Measured	Antibody Screening
Qualitative Measurement	Positive
LOCATION OF DATA	Figure 2
**IMMUNE RESPONSE - B Cell Response Data 2 for EPITOPE 2**	
**IMMUNIZATION**	
**Immunized Species**	
Species	Oryctolagus cuniculus
Strain / Ethnicity	New Zealand
Disease Name	Healthy

Immunization category	Administration
**Immunogen**	
Immunogen Type	Epitope
**Carrier / Vector**	
Carrier Name	Bovine serum ablbumin
Source Species	Bos taurus
**In vivo Immunization**	
Formulation	Peptide emulsion supplemented with Mycobacterium tuberculosis
Adjuvant(s)	Freund's complete; Freund's incomplete (IFA);
Administration Route	Subcutaneous (s.c)
Dose Schedule	Rabbits were injected multiple times on the back. Three weeks later, booster injections were administered with Freund's incomplete adjuvant
**ASSAY**	
**Antibody**	
Antibody Name	anti-N-protein
Antibody Type	Polyclonal
Source Species	Oryctolagus cuniculus
Strain	New Zealand
Isotype	IgG
**Antigen**	
Antigen Type	Epitope
**Carrier / Vector**	
Carrier Name	Bovine serum albumin
Source Species	Bos taurus
**Assay Information**	
Materials Assayed	serum
Assay Type	Western Blot (Immunoblot)
Type of Response Measured	Antibody Screening
Qualitative Measurement	Positive
LOCATION OF DATA	Table 1
COMMENTS ON ASSAY	BSA preabsorption and BSA antigen were conducted as controls with negative responses.
**IMMUNE RESPONSE - B Cell Response Data 3 for EPITOPE 2**	
**IMMUNIZATION**	
**Immunized Species**	
Species	Homo sapiens
Disease Name	SARS
Disease Stage	Unknown
Immunization category	Natural Infection or Exposure
**Immunogen**	
Immunogen Type	Source Species
Source Species	SARS coronavirus
**ASSAY**	
**Antibody**	
Antibody Name	anti-N-protein
Antibody Type	Polyclonal
Source Species	Homo sapiens
Isotype	IgG
**Antigen**	
Antigen Type	Epitope
**Carrier / Vector**	
Carrier Name	Bovine serum albumin

Source Species	Bos taurus
**Assay Information**	
Materials Assayed	serum
Assay Type	Western Blot (Immunoblot)
Type of Response Measured	Antibody Screening
Qualitative Measurement	Negative
Number of Subjects Tested	9
Number of Subjects Responded	0
LOCATION OF DATA	Table 2
