# Supplementary material for: An ontology for immune epitopes: application to the design of a broad scope database of immune reactivities
Source: Immunome Res. 2005 Sep 20;1:2. doi: 10.1186/1745-7580-1-2 (PMC1287064; doi:10.1186/1745-7580-1-2)
Supplement: Additional File 2 — IEDB Data Dictionary v12-5. The Data Dictionary contains the textual overview description and a listing of fields that are captured for IEDB. [file 1745-7580-1-2-S2.pdf]

# IEDB Data Dictionary

**IMPORTANT NOTES: Please read this sheet before reviewing the Data Dictionary**

**This workbook contains two worksheets. The first worksheet provides an overview and lists the minimum fields required to complete one valid epitope entry. The second worksheet lists the data dictionary defined for IEDB. The IEDB Data Dictionary lists all the fields along with their interpretation made for the project. The data dictionary also provides the data type for each field with some sample values. If the values for a field come from a controlled list of selection, it is indicated by the symbol (▼) in the dropdown column. Red colored text is used to indicate main classes under which various sub classes and/or fields are grouped. Classes are grouping of fields by their natural relationship. The final two columns (Data Availability and Comments) are specifically added to be used by large scale epitope discovery team members and other database users for providing input to the IEDB program. Contact the IEDB team for any questions or suggestions.**

## Required Fields

**The following set of fields is the minimum required fields to be completed before an epitope record can be added to the database. Twenty six fields constitute five required fields category. Essentially one set from each category has to be filled out. These fields are highlighted in yellow in the data dictionary**

| # | | | Section Classification | Field Name | Comments |
|---|---|---|---|---|---|
| **1** | **a** | | Reference - Journal Article | PubMed ID | At least one set of fields from Category # 1 (1a, 1b or 1c) has to be filled out. |
| | **b** | **i** | Reference - Submission | Author(s) | |
| | | **ii** | Reference - Submission | Affiliation(s) | |
| | **c** | | Reference - Patents | Patent Publication Number | |
| **2** | **a** | | Epitope Structure | Linear Sequence | At least one of the three fields from Category # 2 (2a, 2b or 2c) has to be filled out. |
| | **b** | | Epitope Structure | SMILES Structure | |
| | **c** | | Epitope Structure | Conformational Sequence | |
| **3** | **a** | | Epitope Structure | Epitopic Region / Domain | Mandatory field. This boolean field indicates whether the epitope that is captured is a minimal epitope or contained within a region / domain. |
| **4** | **a** | | Epitope Source | Antigen Nature | At least one of the seven fields from Category # 4 (4a, 4b, 4c, 4d, 4e, 4f or 4g) has to be filled out. If the value of Natural Antigen, which is a boolean field, is 'no', all other Epitope-Source [illegible] |
| | **b** | | Epitope Source | Source Species | |
| | **c** | | Epitope Source | Gene Name | |
| | **d** | | Epitope Source | Protein Name | |

| | | | | | |
|---|---|---|---|---|---|
| | e | | Epitope Source | GenBank ID | fields are ignored. |
| | f | | Epitope Source | Swiss Prot ID | |
| | g | | Epitope Source | PDB ID | |
| **5** | **a** | **i** | MHC Binding | MHC Allele | At least one set of fields from Category # 5 (5a, 5b, 5c, 5d) has to be filled out. All the fields in a subsection has to be filled out if that subsection is selected. For example, if 5a is chosen, all three fields (5a-i,ii,iii) have to be filled out. The following fields - Assay Type, and Qualitative Measurement, can be entered as "Unknown" if the data is unavailable. It's anticipated that most of the data imports from other existing databases might not have the assay related fields. |
| | | **ii** | MHC Binding | Assay Type | |
| | | **iii** | MHC Binding | Qualitative Measurement | |
| | **b** | **i** | Naturally Processed Ligands | MHC Allele | |
| | | **ii** | Naturally Processed Ligands | Assay Type | |
| | | **iii** | Naturally Processed Ligands | Qualitative Measurement | |
| | **c** | **i** | T Cell Response - Assay | MHC Allele | |
| | | **ii** | T Cell Response - Assay | Assay Type | |
| | | **iii** | T Cell Response - Assay | Qualitative Measurement | |
| | **d** | **i** | B Cell Response - Assay | Assay Type | |
| | | **ii** | B Cell Response - Assay | Qualitative Measurement | |

# IEDB Data Dictionary

| Data Field # | **REFERENCE – Journal Article** | **Reference is a source from which Epitope and its related information are extracted. Journal Article refers to manuscripts published in Journals.** | Sample Values | Data Type | Drop down | Data Availability | Comments |
|---|---|---|---|---|---|---|---|
| 1 | PubMed ID | Unique identifier for records available through PubMed Search Interface. PubMed, a service of the National Library of Medicine, includes over 15 million citations for biomedical articles back to the 1950's. These citations are from MEDLINE and additional life science journals | 10562307 | Number | | | |
| 2 | Journal Title | Title of journal containing the article | J Clin Invest | Varchar2(200) | | | |
| 3 | Journal ISSN | ISSN of Journal in which article is present. ISSN (International Standard Serial Number) is an eight-digit number which identifies periodical publications. | 0021-9738 | Varchar2(15) | | | |
| 4 | Journal Volume | Volume number of journal containing the article | 104 | Varchar2(15) | | | |
| 5 | Journal Issue | Issue number of journal containing the article | 10 | Number | | | |
| 6 | Journal Date | Publication date of the article in Journal | Nov 1999 | Date | | | |
| 7 | Article Title | Title of the article | Naturally processed and presented epitopes of the islet cell autoantigen IA-2 eluted from HLA-DR4 | Varchar2(400) | | | |
| 8 | Article Priority | Priority flag if article contains epitope data related to A-C class pathogens | No | Varchar2(1) | | | |
| 9 | Pages | Page numbers (start and end) of article in the Journal | 1449-57 | Varchar2(24) | | | |
| 10 | Authors | Name of author(s) listed in the article | Peakman M, Stevens EJ, Lohmann T, Alexander A, Tomlinson AJ, Trucco M, Gorga JC, Chicz RM | Varchar2(300) | | | |
| 11 | Affiliations | Institution(s) to which author(s) belong. | Department of Immunology, Denmark Hill Campus, London SE5 | Varchar2(300) | | | |
| 12 | MeSH List | List of MeSH terms in article. MeSH stands for Medical Subject Headings. MeSH is NLM's controlled vocabulary used for indexing articles for MEDLINE/PubMed. MeSH terminology provides a consistent way to retrieve information that may use different terminology for the same concepts. | Amino Acid Sequence, Antigen-Presenting Cells, Arthritis, Rheumatoid | Varchar2(400) | | | |
| 13 | Chemical List | List of Chemicals referenced in the article | HLA-DR4 Antigen, Peptide Fragments, IA-2 protein | Varchar2(240) | | | |
| 14 | Keywords | Keywords listed by the author(s) of the article. | Naturally processed, epitopes | Varchar2(2000) | | | |
| 15 | Abstract | Short summary placed prior to the introduction, often with different line justification (block quote) from the rest of the article, used to help readers determine the purpose of the article | | Varchar2(4000) | | | |
| 16 | Comment | Comments on article supplied by curator, specifically if certain data is in supplementary materials of the article | | Varchar2(2000) | | | |

| Data Field # | REFERENCE –Submission | Submission refers to epitope and its related information submitted to IEDB. Data from Large scale antibody and T cell epitope discovery contracts and those transferred from other websites fall under this section. | Sample Values | Data Type | Drop down | Data Availa bility | Comments |
|---|---|---|---|---|---|---|---|
| 17 | Submission ID | Unique identifier of submission as generated by system | 84823746 | Number | | | |
| 18 | Date | Submission date generated by system | 12/7/04 | Date | | | |
| 19 | Title | Optional title entered by submitting author(s) | Identification of Class I / II restricted epitopes from Variola and Vaccinia Viruses | Varchar2(400) | | | |
| 20 | Priority | Priority flag if reference contains epitope data related to A-C class pathogens | Yes | Varchar2(5) | | | |
| 21 | Submitter Name | Name of person(s) who submits the epitope and its related data to IEDB | Valerie Pasquetto | Varchar2(85) | | | |
| 22 | Author(s) | Name of author(s) to be listed in citations regarding the specific submission | Oseroff C, Pasquetto V, Sette A | Varchar2(300) | | | |
| 23 | Affiliation(s) | Institution(s) to which author(s) belong | La Jolla Institute for Allergy and Immunology | Varchar2(300) | | | |
| 24 | Keywords | Keywords related to the submission, entered by the submitter | Vaccinia Virus, A C pathogenss | Varchar2(2000) | | | |
| 25 | Abstract | Abstract entered by submitter(s) | | Varchar2(4000) | | | |

| Data Field # | REFERENCE – Patents | Issued or pending patents that contain epitope and its related information. | Sample Values | Data Type | Drop down | Data Availa bility | Comments |
|---|---|---|---|---|---|---|---|
| 26 | Patent Publication Number | Unique identifier for patent as assigned by USPTO or WO | 6,602,510 | Varchar2(35) | | | |
| 27 | Publication Date | Date of patent official publication | Dec 2000 | Date | | | |
| 28 | Title | Official patent title | HLA class I A2 tumor associated antigen peptides and vaccine compositions | Varchar2(400) | | | |
| 29 | Priority | Priority flag is patent contains data on A-C class pathogens | No | Varchar2(5) | | | |
| 30 | Inventor(s) | Inventor(s) of patented item | Fikes JD, Sette A, Sidney J, Southwood S, Celis E, Keogh E, Chesnut R | Varchar2(200) | | | |
| 31 | Assignee | Name of individual or entity to which patent ownership was assigned to at the time of patent issue | Epimmune Inc | Varchar2(200) | | | |
| 32 | Keyword(s) | Keywords related to patent article, added by curator | HLA, epitope, tumor, vaccine | Varchar2(2000) | | | |
| 33 | Abstract | Name of individual or entity to which patent ownership was assigned to at the time of patent issue | | Varchar2(4000) | | | |

| Data Field # | | | | | | EPITOPE – STRUCTURE | **An epitope is defined as the chemical structure recognized by antigen specific receptors of the immune system (antibodies and/or T cell receptors). In the case of most T cell epitopes, the epitope is defined as the structure that is presented in association with specific MHC molecules, and is bound by the variable regions of specific TCRs. Likewise, a B cell epitope is any structure bound by antibodies (monoclonal or polyclonal) through their variable regions. Both linear and conformational epitopes are considered within the scope of the database. Data relating to MHC bound peptides is also included even in the absence of available T cell recognition data. The Epitope Structure captures the physical and chemical features of an epitope** | Sample Values | Data Type | Drop down | Data Availability | Comments |
|---|---|---|---|---|---|---|---|---|---|---|---|---|
| | 34 | | | | | Epitope Name | Name of the epitope as used in the reference | NP 218-226 | Varchar2(200) | | | |
| | 35 | | | | | Chemical Type | Categorization of epitope compound as lipid, carbohydrate, DNA, RNA, peptide, organic, inorganic, etc. | Peptide, Lipid | Varchar2(35) | ▼ | | |
| | 36 | | | | | Continuous Epitope | Discontinuous epitopes, on a protein antigen are formed from several separate regions in the primary sequence of a protein brought together by protein folding. Antibodies that bind discontinuous epitopes bind only native folded proteins. | Continuous/ Discontinuous | Varchar2(85) | ▼ | | |
| | 37 | | | | | SMILES Structure | SMILES notation for 2-D structure of epitope. SMILES stand for Simplified Molecular Input Line Entry Specification. SMILES is widely used as a general-purpose chemical nomenclature and data exchange format | CN1CCC[C@H]1c2cccnc2 (*SMILES structure for Nicotine* ) | Varchar2(3500) | | | |
| | 38 | | | | | Linear Sequence | Linear sequence of epitope amino acids, if epitope is a peptide. Linear sequence field also captures continuous epitopes that has a conformation. Other chemical structures are captured using SMILES structure. | AYERMCNIL | Varchar2(50) | | | |
| | 39 | | | | | Conformational Sequence | Sometimes epitopes that contain conformational determinant consists of amino acid residues that are not contiguous within the protein, but are brought together on the surface by folding of the protein. In such cases the discontinuous amino acid sequence constituting the epitope are entered in alphanumeric or position notation. | A21,Y22,E33,R34,M35,S80,N81,I82,L83 | Varchar2(2000) | | | |
| | 40 | | | | | Modification Type | Modification Type captures post-translational modification of the epitope and other modifications including formylation, oxidation of cystine, etc. Post-translational modification is the enzymatic processing of a polypeptide chain after translation from messenger RNA and after peptide bond formation has occurred. IEDB conforms to the SWISS-PROT standard list for post-translational modification categories but supplemented with additional ones as appropriate. | Sulfhydryl modification | Varchar2(85) | ▼ | | |
| | 41 | | | | | Modified Sequence | Indicates the residues that are modified by the modification type. Single letter amino acid code of the residue is used followed by the it's position within the epitope. | S6 | Varchar2(4000) | | | |
| | 42 | | | | | Author Identified Mimotopes | Mimotope is a structure unrelated to the antigen or immunogen that is recognized by the same antigen receptor. If yes, the specific mimotope information is captured in the comments. | Yes / No | Varchar2(85) | | | |
| | 43 | | | | | Comments | Curator added comments | | Varchar2(2000) | | | |

| | | | | | | | | | | | | |
|---|---|---|---|---|---|---|---|---|---|---|---|---|
| | 44 | | | | | Epitopic Region / Domain | If the exact location of the epitope in its source is unknown, but the region or domain in which it may be contained is less than 50 residues in length or 5000 daltons in molecular weight, the region or domain is captured as an epitope with this field specified as "Yes". When the value is "Yes", the epitope structure fields does not represent the epitope, but rather the region in which it is present. | Yes / No | Varchar2(1) | | | |
| **Data Field #** | | | | | | **EPITOPE – SOURCE** | **Epitope Source refers to the phylogenetic or chemical source of the epitope.** | **Sample Values** | **Data Type** | **Drop down** | **Data Availability** | **Comments** |
| | 45 | | | | | Source Species | Source species of antigen containing the epitope, selected from a hierarchical list of species from NCBI taxonomy database . | Influenza A Virus | Varchar2(150) | ▼ | | |
| | 46 | | | | | Species Strain | Strain of epitope source species either as specified in NCBI Taxonomy database or recorded by the curator. | A/PR/84 | Varchar2(85) | | | |
| | 47 | | | | | Chemical Type | Categorization of antigen compound as protein, lipid, carbohydrate, DNA, RNA, organic, inorganic, etc. | Protein | Varchar2(35) | ▼ | | |
| | 48 | | | | | Gene Name | Name of the Gene coding for the epitope | NP | Varchar2(200) | | | |
| | 49 | | | | | Protein Name | Name of the Protein containing epitope | Nucleoprotein (NP) | Varchar2(200) | | | |
| | 50 | | | | | Swiss-Prot ID | Unique identifier of protein sequence in Swiss-Prot database. If the protein sequence identifier is not provider by the author, curator finds them, when applicable. | Q07539 | Varchar2(35) | | | |
| | 51 | | | | | Epitope Starting Position | Starting position of continuous epitope in the source antigen sequence as mentioned in the reference. Position of residues in discontinuous epitopes are captured in Conformation Sequence field. | 218 | Number | | | |
| | 52 | | | | | Epitope Ending Position | Ending position of the continuous epitope in the source antigen sequence as mentioned in the reference. Position of residues in discontinuous epitopes are captured in Conformation Sequence field. | 226 | Number | | | |
| | 53 | | | | | Epitope Swiss-Prot / GenBank Positions | Starting and Ending Position of epitope in source antigen. Entered only if the author specified epitope positions is different from those in the protein sequence identified by the curator | 219-227 | Varchar2(35) | | | |
| | 54 | | | | | GenBank ID | Unique identifier of gene sequence in GenBank database | M23976 | Varchar2(35) | | | |
| | 55 | | | | | PDB ID | Identification of source if structure is present in PDB. | 1AIL | Varchar2(35) | | | |
| | 56 | | | | | Epitope PDB positions | List of PDB positions of residues representing epitope | D20-D22, D129-D132, C25-C29, C126-C127, C20-C22 | Varchar2(200) | | | |
| | 57 | | | | | Functional multimer | Number of monomers if the antigen structure is a functional multimer. | 1 | Number | | | |
| | 58 | | | | | Epitope Source Nature | Indicates whether the antigen is a natural or artificial. Natural here refers to things that exist in nature. | Natural Antigen / Artificial Antigen | Varchar2(85) | ▼ | | |
| | 59 | | | | | Comments | Curator added comments | | Varchar2(2000) | | | |

| Data Field # | MHC BINDING | MHC Binding captures the details of MHC molecules and epitope binding information with the MHC molecule along with Epitope-MHC complex details, if available. | Sample Values | Data Type | Drop down | Data Availability | Comments |
|---|---|---|---|---|---|---|---|
| | **Binding Details** | This sub section captures the MHC binding details. | | | | | |
| 60 | Source Species of MHC | Species of the MHC molecule, selected from a hierarchical list of species from NCBI taxonomy database . | Human | Varchar2(150) | ▼ | | |
| 61 | Species Strain | Strain of MHC source species either as specified in NCBI Taxonomy database or recorded by the curator. | | Varchar2(85) | | | |
| 62 | MHC Class | MHC is broadly divided into class I and class II. There are also non-classical MHC molecules. In general, MHC class I molecules present peptides generated in the cytosol to CD8 T cells and MHC class II molecules present peptides to CD4 T cells. | I | Varchar2(35) | ▼ | | |
| 63 | MHC Allele | Alleles are mutually exclusive forms of the same gene, occupying the same locus on homologous chromosomes within a species, and governing the same biochemical and developmental process.IEDB conforms to HLA workshop nomeclature for the HLA alleles and Immuno Polymorphism Database (IPD) for other MHC alleles, where applicable.This field captures the MHC Allele that epitope binds to. | HLA A*0201 | Varchar2(35) | ▼ | | |
| 64 | Assay type | Name of the experimental setup used to measure epitope binding to MHC molecule | Competition Assay / Stabilization Assay | Varchar2(50) | ▼ | | |
| 65 | Response Measured | Type of parameter measured using the assay. | IC 50 / EC50 | Varchar2(85) | ▼ | | |
| 66 | Qualitative Measurement | Qualitative assessment of the binding value measured using the assay as reported in the reference. | Positive / Negative | Varchar2(35) | ▼ | | |
| 67 | Measurement Inequality | Inequality of the quantitative measurement captured. By default, measurement inequality is "=". | > / < / >= / <= / = | Varchar2(5) | ▼ | | |
| 68 | Quantitative Measurement | Actual numerical value measured using the assay as reported in the reference. | 10 | Number | | | |
| 69 | Units | Standard quantities of measurement which are specific to a type of measurement. | nM | Varchar2(15) | ▼ | | |
| 70 | Location of Data | Free text mentioning the location of Assay related data in the reference. | Table 4, Page 324 | Varchar2(35) | | | |
| 71 | Comments | Curator added comments | | Varchar2(2000) | | | |
| | **Chain #1** | MHC molecules are comprised of two polypeptide chains – alpha chain and beta chain or beta-2-microglobulin. This sub section captures fields related to alpha chain | | | | | |
| 72 | Chain type | Name of MHC polypeptide chain | Class I Alpha | Varchar2(35) | ▼ | | |
| 73 | Gen Bank ID | GenBank ID of MHC Chain. It's the unique identifier of gene sequence in GenBank database | K02883 | Varchar2(35) | | | |
| 74 | Swiss Prot ID | Swiss-Prot ID of MHC Chain. It's the unique identifier of protein sequence in SWISS-PROT database | P01892 | Varchar2(35) | | | |
| 75 | PDB ID | It's a unique PDB identifier of molecular chain representing MHC chain. | 1AKJ | Varchar2(35) | | | |
| | **Chain #2** | This sub section captures fields related to beta chain or beta-2-microglobulin. | | | | | |
| 76 | Chain type | Name of MHC polypeptide chain | Beta-2-microglobulin | Varchar2(35) | ▼ | | |
| 77 | Gen Bank ID | GenBank ID of MHC Chain. It's the unique identifier of gene sequence in GenBank database | M17987 | Varchar2(35) | | | |

| | | | | | | | | | | | | |
|---|---|---|---|---|---|---|---|---|---|---|---|---|
| | | 78 | | | | Swiss Prot ID | Swiss-Prot ID of MHC Chain. It's the unique identifier of protein sequence in SWISS-PROT database | P61769 | Varchar2(35) | | | |
| | | 79 | | | | PDB ID | It's a unique PDB identifier of molecular chain representing MHC chain. | 1BOR | Varchar2(35) | | | |
| | | | | | | **Epitope-MHC Complex** | This sub section captures all related structure fields if structure information for epitope-MHC complex is available | | | | | |
| | | 80 | | | | Complex PDB ID | Unique four-letter PDB identifier of molecular structure representing epitope-MHC complex. | 1ABC | Varchar2(35) | | | |
| | | 81 | | | | Antigen residues interacting with MHC | List of epitope residues interacting with MHC | H 103, E 120 | Varchar2(200) | | | |
| | | 82 | | | | MHC residues interacting with antigen | List of MHC residues interacting with epitope | F 111, S 222 | Varchar2(200) | | | |
| | | 83 | | | | Antigen Contact Area | Contact area of epitope interacting with MHC | 800 | Number | | | |
| | | 84 | | | | MHC Contact Area | Contact area of MHC interacting with epitope | 760 | Number | | | |
| | | 85 | | | | Interacting atom pairs | Pairs of atoms involved in the interaction between epitope and MHC | A 103 OG1, B 111 N3 | Varchar2(2000) | | | |
| | | 86 | | | | Allosteric effect | This Boolean field indicates whether an allosteric effect has taken place. Allosteric effect is of or involving a change in the shape and activity of a molecule's structure that results from molecular binding with a regulatory substance at a site other than the active one where changes take place. | No / Yes | Varchar2(1) | | | |
| | | 87 | | | | Cofactor/Effector Name | The name of cofactor changing the epitope-MHC interaction activity if such a change has taken place. | Human Membrane cofactor protein | Varchar2(200) | | | |
| | 88 | | | | | **Comments** | Curator added comments | | Varchar2(2000) | | | |

| **Data Field #** | | | | | | **PEPTIDE ELUTION DATA** | **Peptide Elution Data captures information relating to the determination of epitopes that are naturally bound by MHC molecules. Antigen-presenting cells process antigens and present peptide epitopes complexed with MHC molecules.** | **Sample Values** | **Data Type** | **Drop down** | **Data Availability** | **Comments** |
|---|---|---|---|---|---|---|---|---|---|---|---|---|
| | | | | | | **Antigen Presentation** | This sub section captures information about the antigen presenting cells. These are cells that process and present eptiopes to effector cells. | | | | | |
| | | | | | | **MHC** | This section captures the details of MHC molecules presented by the Antigen presenting cells | | | | | |
| | | | | 89 | | MHC Class | Classification of Major Histocompatibility Complex (MHC). The MHC is a cluster of genes important in immune recognition and signaling between cells of the immune system. | I | Varchar2(35) | ▼ | | |
| | | | | 90 | | MHC Allele | Alleles are mutually exclusive forms of the same gene, occupying the same locus on homologous chromosomes within a species, and governing the same biochemical and developmental process.IEDB conforms to HLA workshop nomeclature for the HLA alleles and Immuno Polymorphism Database (IPD) for other MHC alleles, where applicable.This field captures the MHC Allele that epitope binds to. | HLA A*0201 | Varchar2(35) | ▼ | | |

| | | | | | | | | | | | | |
|---|---|---|---|---|---|---|---|---|---|---|---|---|
| | | | | | | **MHC Chain #1** | MHC molecules are comprised of two polypeptide chains – alpha chain and beta chain or beta-2-microglobulin. This sub section captures fields related to alpha chain | | | | | |
| | | | | | 91 | Chain type | Name of MHC polypeptide chain | Class I Alpha | Varchar2(35) | ▼ | | |
| | | | | | 92 | Gen Bank ID | GenBank ID of MHC Chain. It's the unique identifier of gene sequence in GenBank database | K02883 | Varchar2(35) | | | |
| | | | | | 93 | Swiss Prot ID | Swiss-Prot ID of MHC Chain. It's the unique identifier of protein sequence in SWISS-PROT database | P01892 | Varchar2(35) | | | |
| | | | | | 94 | PDB ID | It's a unique PDB identifier of molecular chain representing MHC chain. | 1AKJ | Varchar2(35) | | | |
| | | | | | | **MHC Chain #2** | This sub section captures fields related to beta chain or beta-2-microglobulin. | | | | | |
| | | | | | 95 | Chain type | Name of MHC polypeptide chain | Beta-2-microglobulin | Varchar2(35) | ▼ | | |
| | | | | | 96 | Gen Bank ID | GenBank ID of MHC Chain. It's the unique identifier of gene sequence in GenBank database | M17987 | Varchar2(35) | | | |
| | | | | | 97 | Swiss Prot ID | Swiss-Prot ID of MHC Chain. It's the unique identifier of protein sequence in SWISS-PROT database | P61769 | Varchar2(35) | | | |
| | | | | | 98 | PDB ID | It's a unique PDB identifier of molecular chain representing MHC chain. | 1BOR | Varchar2(35) | | | |
| | | | | | | **Source Species** | This sub section captures the species information the antigen presenting cells, which express the MHC molecules. | | | | | |
| | | | | 99 | | Species | Source species of the antigen presenting cells, selected from a hierarchical list of species from NCBI taxonomy database and ICTV | Human | Varchar2(150) | ▼ | | |
| | | | | 100 | | Species Strain | Strain of the species either as specified in NCBI Taxonomy database or recorded by the curator. | | Varchar2(85) | | | |
| | | | | 101 | | Sex | Sex of the individuals tested | Male | Varchar2(10) | ▼ | | |
| | | | | 102 | | Age | Age of the individuals tested | 20 | Varchar2(35) | | | |
| | | | | 103 | | MHC types present | List of MHC Alleles expressed by the antigen presenting cells | A*0201, B*1801 | Varchar2(85) | | | |
| | | | | 104 | | Disease Name | Disease state of the individual(s) as a result of immunization or the disease the individual(s) are associated with. IEDB conforms to International Classification of Diseases (ICD-10), if available | HCV infection | Varchar2(200) | ▼ | | |
| | | | | 105 | | Disease Stage | Disease stage of the individual(s) at the stage when effector cells are assayed for response. | Chronic | Varchar2(85) | ▼ | | |
| | | | | | | **Antigen Presenting Cells** | This sub section stores the name of antigen presenting cells and its origin | | | | | |
| | | | | 106 | | Tissue or Cell Type | Name of the tissue or cell type of the antigen presenting cells | PBMC / Spleen / etc | Varchar2(85) | ▼ | | |
| | | | | 107 | | Origin | Origin of the cells (ex vivo, in vivo, in vitro, cell lines, clones) | ex vivo / Cell line / clone / etc | Varchar2(85) | ▼ | | |
| | | | | | | **Antigen** | Substance that is used to detect the immune response elicited by the immunization, and from which the epitope presented by the MHC molecule, is derived. In this context the antigen is the molecular structure that contains the epitope, and from which epitope is derived as a result of natural processing. | | | | | |
| | | | 108 | | | Antigen Type | This field indicates if Antigen is same as one of the following - epitope, source protein of the epitope, source species of the epitope, or peptide containing the epitope. If antigen is none of these choices, other antigen fields that are listed below are filled out. | Epitope / Source Protein / Source Species / Peptide containing Epitope | Varchar2(85) | ▼ | | |
| | | | 109 | | | Antigen Name | Name of antigen as mentioned in the reference | NP 218-226 | Varchar2(200) | | | |
| | | | 110 | | | Gene Name | Gene name coding for the antigen used in the assay | NP | Varchar2(200) | | | |

| | | | | | | | | | | | | |
|---|---|---|---|---|---|---|---|---|---|---|---|---|
| | | | 111 | | | Chemical Type | Categorization of antigen compound as protein, lipid, carbohydrate, DNA, RNA, organic, inorganic, etc. | Peptide, Lipid | Varchar2(35) | ▼ | | |
| | | | 112 | | | Source Species | Source Species that produced the antigen used in the assay, selected from a hierarchical list of species from NCBI taxonomy database | Influenza A Virus | Varchar2(150) | ▼ | | |
| | | | 113 | | | Species Strain | Strain of the species either as specified in NCBI Taxonomy database or recorded by the curator. | | Varchar2(85) | | | |
| | | | 114 | | | SMILES Structure | SMILES notation for 2-D structure. SMILES stand for Simplified Molecular Input Line Entry Specification. SMILES is widely used as a general-purpose chemical nomenclature and data exchange format | | Varchar2(3500) | | | |
| | | | 115 | | | Sequence | Linear sequence of antigen amino acids. Other chemical structures will be captured using SMILES structure. | AYERMCNIL | Varchar2(500) | | | |
| | | | 116 | | | GenBank ID | Unique identifier of antigen used in the assay in GenBank database | K02883 | Varchar2(35) | | | |
| | | | 117 | | | Swiss Prot ID | Unique identifier of sequence of antigen used in the assay in Swiss-Prot database | P01892 | Varchar2(35) | | | |
| | | | 118 | | | PDB ID | Unique PDB identifier of molecular chain representing antigen used in the assay | 1AKJ | Varchar2(35) | | | |
| | | | | | | **Carrier / Vector** | Carrier is a molecular structure that is not normally associated with the epitope. The epitope is covalently linked in vitro or in vivo, thereby in general modulating its immunogenicity, antigenicity or processing. A vector is an entity (virus, bacteria) in which the epitope is incorporated for the purpose of delivering to the cells or organism. | | | | | |
| | | | | 119 | | Carrier Name | Name of carrier as reported in the reference. | MUC1 Glycoprotein B cell Epitope | Varchar2(200) | | | |
| | | | | 120 | | Chemical Type | Categorization of carrier compound as protein, lipid, carbohydrate, DNA, RNA, organic, inorganic, etc. | Peptide | Varchar2(35) | ▼ | | |
| | | | | 121 | | SMILES Structure | SMILES notation for 2-D structure. SMILES stand for Simplified Molecular Input Line Entry Specification. SMILES is widely used as a general-purpose chemical nomenclature and data exchange format | | Varchar2(3500 ) | | | |
| | | | | 122 | | Source Species | Source species from which the carrier is derived. The values are selected from a hierarchical list of species from NCBI taxonomy database and ICTV | Human | Varchar2(150) | ▼ | | |
| | | | | 123 | | Species Strain | Strain of the species if not specified in NCBI Taxonomy database, which usually has strain information for species. | | Varchar2(85) | | | |
| | | | | 124 | | Sequence | Linear sequence of carrier amino acids. Other chemical structures will be captured using SMILES structure. | YKQGGFLGL | Varchar2(4000) | | | |
| | | | | 125 | | GenBank ID | Unique identifier of carrier in GenBank database | X91302 | Varchar2(35) | | | |
| | | | | 126 | | Swiss Prot ID | Unique identifier of carrier sequence in SWISS-PROT database | Q68840 | Varchar2(35) | | | |
| | | | | 127 | | PDB ID | Unique PDB identifier of molecular chain representing carrier | 1CWX | Varchar2(35) | | | |
| | | 128 | | | | **Special Culture Conditions** | Free text capturing any special culture conditions used in the assay | | Varchar2(500) | | | |
| | | | | | | **Assay Information** | This section captures information about the experiment used to detect the immune response of epitopes that are naturally bound by MHC molecules | | | | | |
| | | | 129 | | | Assay Type | Name of the experimental setup used to measure the immune response. | Mass Spectrometry | Varchar2(50) | ▼ | | |
| | | | 130 | | | Response Measured | Type of response measured using the assay. | Presentation of Peptides | Varchar2(85) | ▼ | | |

| | | | | | | | | | | | | |
|---|---|---|---|---|---|---|---|---|---|---|---|---|
| | | | 131 | | | Qualitative Measurement | Qualitative assessment of the value measured using the assay as reported in the reference. | Positive / Negative | Varchar2(35) | ▼ | | |
| | | | 132 | | | Measurement Inequality | Inequality of the quantitative measurement captured. By default, measurement inequality is "=". | > / < / >= / <= / = | Varchar2(5) | ▼ | | |
| | | | 133 | | | Quantitative Measurement | Actual numerical value measured using the assay as reported in the reference. | 1635 | Number | | | |
| | | | 134 | | | Units | Standard quantities of measurement which are specific to a type of measurement. | amu | Varchar2(15) | ▼ | | |
| | | | 135 | | | Location of Data | Free text mentioning the location of Assay related data in the reference. | Figure 2 | Varchar2(35) | | | |
| | | 136 | | | | Comments | Comments entered by Curator related to peptide elution data. | | Varchar2(2000) | | | |

| Data Field # | | | | | | **T-CELL RESPONSE** | **T Cell Response captures all the cell mediated immunity and it's divided into two broad sub sections – Immunization and Assay.** | **Sample Values** | **Data Type** | **Drop down** | **Data Availability** | **Comments** |
|---|---|---|---|---|---|---|---|---|---|---|---|---|
| | | | | | | **IMMUNIZATION** | Immunization describes how the immune system is exposed to an immunogen. This sub section has categories that capture information about the immunized species, immunogen and in vitro or in vivo immunization. | | | | | |
| | | | | | | **Immunized Species** | Information about the source species that is being immunized | | | | | |
| | | | 137 | | | Species | Species that received immunization by immunogen, selected from a hierarchical list of species from NCBI taxonomy database and ICTV | Mus musculus (Mouse) | Varchar2(150) | ▼ | | |
| | | | 138 | | | Strain / Ethnicity | Genetic variant or an inbred line of a higher organism. The values for strain are either as specified in NCBI Taxonomy database or recorded by the curator. In case of human this field store information relating to the ethnicity individuals studied, conforming to HLA workshop conventions, if applicable. Ethnicity relates to large groups of people classed according to common racial, national, tribal, religious, linguistic, or cultural origin or background. | Balb/c | Varchar2(85) | | | |
| | | | 139 | | | Sex | Sex of the individuals tested | Female | Varchar2(10) | | | |
| | | | 140 | | | Age | Age of the individuals tested | 6-8weeks | Varchar2(35) | | | |
| | | | 141 | | | MHC types present | List of MHC Alleles expressed in the immunized species | H2-d | Varchar2(85) | ▼ | | |
| | | | 142 | | | Disease Name | Disease state of the individual(s) as a result of immunization or the disease the individual(s) are associated with. IEDB conforms to International Classification of Diseases (ICD-10), if available | HCV infection | Varchar2(85) | ▼ | | |
| | | | 143 | | | Disease Stage | Disease stage of the individual(s) at the stage when effector cells are assayed for response. | Chronic | Varchar2(85) | ▼ | | |
| | | | 144 | | | Immunization Category | This field captures how the species was immunized. | Natural Infection or exposure, Administration, etc | Varchar2(85) | ▼ | | |
| | | | | | | **Immunogen** | Substance that is capable of inducing an immune response (antibody response or cell mediated immunity) and contains the epitope. | | | | | |

| | | | | | | | | | | | | |
|---|---|---|---|---|---|---|---|---|---|---|---|---|
| | | | 145 | | | Immunogen Type | This field indicates if immunogen is same as one of the following - epitope, source protein of the epitope, source species of the epitope, or peptide containing the epitope. If immunogen is none of these choices, other imunogen fields that are listed below are filled out. | Epitope / Source Protein / Source Species / Peptide containing Epitope | Varchar2(85) | ▼ | | |
| | | | 146 | | | Immunogen Name | Name of immunogen as mentioned in the reference. | HCV | Varchar2(200) | | | |
| | | | 147 | | | Chemical Type | Categorization of immunogen compound as protein, lipid, carbohydrate, DNA, RNA, organic, inorganic, etc. | DNA / RNA | Varchar2(35) | ▼ | | |
| | | | 148 | | | SMILES Structure | SMILES notation for 2-D structure. SMILES stand for Simplified Molecular Input Line Entry Specification. SMILES is widely used as a general-purpose chemical nomenclature and data exchange format | | Varchar2(3500 ) | | | |
| | | | 149 | | | Source Species | Source species that produces the immunogen, selected from a hierarchical list of species from NCBI taxonomy database and ICTV | Hepatitis C virus | Varchar2(150) | ▼ | | |
| | | | 150 | | | Species Strain | Strain of the species either as specified in NCBI Taxonomy database or recorded by the curator. | | Varchar2(85) | | | |
| | | | 151 | | | Sequence | Linear sequence of immunogen amino acids. Other chemical structures will be captured using SMILES structure. | | Varchar2(4000) | | | |
| | | | 152 | | | GenBank ID | Unique identifier of immunogen in GenBank database | X91302 | Varchar2(35) | | | |
| | | | 153 | | | Swiss Prot ID | Unique identifier of immunogen sequence in SWISS-PROT database | Q68840 | Varchar2(35) | | | |
| | | | 154 | | | PDB ID | Unique PDB identifier of molecular chain representing immunogen | 1CWX | Varchar2(35) | | | |
| | | | | | | **Carrier / Vector** | Carrier is a molecular structure that is not normally associated with the epitope. The epitope is covalently linked in vitro or in vivo, thereby in general modulating its immunogenicity, antigenicity or processing. A vector is an entity (virus, bacteria) in which the epitope is incorporated for the purpose of delivering to the cells or organism. | | | | | |
| | | | | 155 | | Carrier Name | Name of carrier as reported in the reference. | MUC1 Glycoprotein B cell Epitope | Varchar2(200) | | | |
| | | | | 156 | | Chemical Type | Categorization of carrier compound as protein, lipid, carbohydrate, DNA, RNA, organic, inorganic, etc. | Peptide | Varchar2(35) | ▼ | | |
| | | | | 157 | | SMILES Structure | SMILES notation for 2-D structure. SMILES stand for Simplified Molecular Input Line Entry Specification. SMILES is widely used as a general-purpose chemical nomenclature and data exchange format | | Varchar2(3500 ) | | | |
| | | | | 158 | | Source Species | Source species that produces the carrier, selected from a hierarchical list of species from NCBI taxonomy database and ICTV | Human | Varchar2(150) | ▼ | | |
| | | | | 159 | | Sequence | Linear sequence of carrier amino acids. Other chemical structures will be captured using SMILES structure. | YKQGGFLGL | Varchar2(4000) | | | |
| | | | | 160 | | Species Strain | Strain of the species either as specified in NCBI Taxonomy database or recorded by the curator. | | Varchar2(85) | | | |
| | | | | 161 | | GenBank ID | Unique identifier of carrier in GenBank database | X91302 | Varchar2(35) | | | |
| | | | | 162 | | Swiss Prot ID | Unique identifier of carrier sequence in SWISS-PROT database | Q68840 | Varchar2(35) | | | |
| | | | | 163 | | PDB ID | Unique PDB identifier of molecular chain representing carrier | 1CWX | Varchar2(35) | | | |
| | | | | | | **In-Vitro Immunization** | This sub section capture fields related to immunization that is performed on cells that are not in living organism, as in a test tube. In-Vitro in latin means glass. | | | | | |
| | | | | | | **Cells at the time of Immunization** | This sub section captures the details of cells at the time of immunization. | | | | | |
| | | | | 164 | | Responder Cells | Name of the tissue or cell type that responds to immunization | CTL | Varchar2(85) | ▼ | | |
| | | | | 165 | | Stimulator Cells | Name of the cells that stimulate responders to induce immune response | PBMC | Varchar2(85) | ▼ | | |

| | | | | | | | | | | | | |
|---|---|---|---|---|---|---|---|---|---|---|---|---|
| | | | | 166 | | Restimulation Comments | Comments about the in vitro immunization / restimulation process | | Varchar2(2000) | | | |
| | | | | | | **In-Vivo Immunization** | This sub section captures fields related to immunization that is performed on living organism. | | | | | |
| | | | | | | **Formulation** | The mixture of chemicals and/or biological substances that are not covalently linked with the immunogen, but are co-administered with it. | | | | | |
| | | | | 167 | | Formulation | Physical form of the mixture containing the immunogen | Liquid / Powder / Oil / Aerosol / Other | Varchar2(35) | | | |
| | | | | 168 | | Adjuvant(s) | Adjuvants are substances that are administered with the immunogen to enhance the immune response and are not covalently linked (opposed to a carrier) to the immunogen. | incomplete Freund's adjuvant | Varchar2(400) | ▼ | | |
| | | | | | | **Administration** | Process of immunizing the source species | | | | | |
| | | | | 169 | | Route | Route of administration | Direct Addition / Intravenous / Intraperitoneal / Subcutaneous / etc | Varchar2(35) | ▼ | | |
| | | | | 170 | | Number of Immunization | Captures the administration schedule. | 4 | Varchar2(250) | | | |
| | | 171 | | | | **Comments** | Comments entered by Curator related to Immunization | | Varchar2(2000 ) | | | |
| | | | | | | **ASSAY** | Assay is a quantitative or qualitative evaluation or test of substance used to describe and or measure biological response. This sub section specifically captures T-cell immune responses and has categories that capture information about effector cells, antigen presenting cells, antigen, culture conditions and assay information. | | | | | |
| | | | | | | **Effector Cells** | This sub section captures information about effector cells. In general these cells were stimulated by immunization and have acquired measurable functions as a result. In certain cases, effector function can be assayed from naive (not previously immunized) cells. | | | | | |
| | | | 172 | | | Tissue or Cell Type | Name of the tissue or cell type of effector cells | CTL / Splenocytes | Varchar2(85) | ▼ | | |
| | | | 173 | | | Origin | Origin of the cells (ex vivo, in vivo, in vitro, cell lines, clones) | | | ▼ | | |
| | | | 174 | | | TCR Name | Name of T-cell receptor as reported in the reference | T17T-22 | Varchar2(200) | | | |
| | | | 175 | | | TCR Source Species | Source species of T-cell receptor, selected from a hierarchical list of species from NCBI taxonomy database | | Varchar2(150) | ▼ | | |
| | | | 176 | | | Species Strain | Strain of the species either as specified in NCBI Taxonomy database or recorded by the curator. | | Varchar2(85) | | | |
| | | | | | | **TCR Chain #1** | TCR consists of two chains, alpha and beta, closely associated with the CD3 protein complex. This latter complex is a family of non-polymorphic chains. This sub section captures TCR alpha chain details | | | | | |
| | | | | 177 | | TCR Chain type | Name of TCR Chain Type. | T-cell rececptor alpha chain | Varchar2(35) | ▼ | | |
| | | | | 178 | | Gen Bank ID | GenBank ID of TCR. It’s the unique identifier of gene sequence in GenBank database | AJ416332 | Varchar2(35) | | | |
| | | | | 179 | | Swiss Prot ID | Swiss-Prot ID of TCR. It’s the unique identifier of protein sequence in SWISS-PROT database | Q65ZL6 | Varchar2(35) | | | |
| | | | | 180 | | PDB ID | Unique PDB identifier of molecular chain representing antibody | 1NFD | Varchar2(35) | | | |

| | | | | | | | | | | | | |
|---|---|---|---|---|---|---|---|---|---|---|---|---|
| | | | | | | **TCR Chain #2** | This sub section captures TCR beta chain details | | | | | |
| | | | | 181 | | TCR Chain type | Name of TCR Chain Type. | T-cell receptor beta chain | Varchar2(35) | ▼ | | |
| | | | | 182 | | Gen Bank ID | GenBank ID of TCR. It's the unique identifier of gene sequence in GenBank database | AJ416332 | Varchar2(35) | | | |
| | | | | 183 | | Swiss Prot ID | Swiss-Prot ID of TCR. It's the unique identifier of protein sequence in SWISS-PROT database | P11364 | Varchar2(35) | | | |
| | | | | 184 | | PDB ID | Unique PDB identifier of molecular chain representing antibody | 1NFD | Varchar2(35) | | | |
| | | | | | | **Antigen Presentation** | This sub section captures information about the antigen presenting cells. These are cells that serve as triggers for effector cells to express effector functions. | | | | | |
| | | | | | | **MHC** | This section captures the details of MHC molecules presenting the epitope. | | | | | |
| | | | | 185 | | MHC Class | Classification of Major Histocompatibility Complex (MHC). The MHC is a cluster of genes important in immune recognition and signaling between cells of the immune system. | I | Varchar2(35) | ▼ | | |
| | | | | 186 | | MHC Allele | Alleles are mutually exclusive forms of the same gene, occupying the same locus on homologous chromosomes within a species, and governing the same biochemical and developmental process.IEDB conforms to HLA workshop nomeclature for the HLA alleles and Immuno Polymorphism Database (IPD) for other MHC alleles, where applicable. This field captures the MHC Allele that epitope is restricted to. | HLA A*0201 | Varchar2(35) | ▼ | | |
| | | | | | | **MHC Chain #1** | MHC molecules are comprised of two polypeptide chains – alpha chain and beta chain or beta-2-microglobulin. This sub section captures fields related to alpha chain | | | | | |
| | | | | | 187 | Chain type | Name of MHC polypeptide chain | Class I Alpha | Varchar2(35) | ▼ | | |
| | | | | | 188 | Gen Bank ID | GenBank ID of MHC Chain. It's the unique identifier of gene sequence in GenBank database | K02883 | Varchar2(35) | | | |
| | | | | | 189 | Swiss Prot ID | Swiss-Prot ID of MHC Chain. It's the unique identifier of protein sequence in SWISS-PROT database | P01892 | Varchar2(35) | | | |
| | | | | | 190 | PDB ID | It's a unique PDB identifier of molecular chain representing MHC chain. | 1AKJ | Varchar2(35) | | | |
| | | | | | | **MHC Chain #2** | This sub section captures fields related to beta chain or beta-2-microglobulin. | | | | | |
| | | | | | 191 | Chain type | Name of MHC polypeptide chain | Beta-2-microglobulin | Varchar2(35) | ▼ | | |
| | | | | | 192 | Gen Bank ID | GenBank ID of MHC Chain. It's the unique identifier of gene sequence in GenBank database | M17987 | Varchar2(35) | | | |
| | | | | | 193 | Swiss Prot ID | Swiss-Prot ID of MHC Chain. It's the unique identifier of protein sequence in SWISS-PROT database | P61769 | Varchar2(35) | | | |
| | | | | | 194 | PDB ID | It's a unique PDB identifier of molecular chain representing MHC chain. | 1BOR | Varchar2(35) | | | |
| | | | | | | **Source Species** | This sub section captures the species information of the antigen presenting cells | | | | | |
| | | | | 195 | | Autologous or Syngeneic Antigen Presenting Cells? | This field indicates if the antigen presenting cells are autologous (from the same individual) or syngeneic (from genetically identical individuals like inbred strain of laboratory animals) with the effector cells, which are obtained from the immunization process. If entered yes, other source species fields are not entered. | Yes / No | Varchar2(1) | | | |
| | | | | 196 | | Species | Source species of the antigen presenting cells, selected from a hierarchical list of species from NCBI taxonomy database | Human | Varchar2(150) | ▼ | | |

| | | | | | | | | | | | | |
|---|---|---|---|---|---|---|---|---|---|---|---|---|
| | | | | 197 | | Strain / Ethnicity | Genetic variant or an inbred line of a higher organism. The values for strain are either as specified in NCBI Taxonomy database or recorded by the curator. In case of human this field store information relating to the ethnicity individuals studied, conforming to HLA workshop conventions, if applicable. Ethnicity relates to large groups of people classed according to common racial, national, tribal, religious, linguistic, or cultural origin or background. | Caucasian | Varchar2(85) | | | |
| | | | | 198 | | Sex | Sex of the individuals tested | Male | Varchar2(10) | ▼ | | |
| | | | | 199 | | Age | Age of the individuals tested | 20 | Varchar2(35) | | | |
| | | | | 200 | | MHC types present | List of MHC Alleles expressed by the antigen presenting cells | A*0201, B*1801 | Varchar2(85) | ▼ | | |
| | | | | 201 | | Disease Name | Disease state of the individual(s) as a result of immunization or the disease the individual(s) are associated with. IEDB conforms to International Classification of Diseases (ICD-10), if available | HCV infection | Varchar2(85) | ▼ | | |
| | | | | 202 | | Disease Stage | Disease stage of the individual(s) at the stage when effector cells are assayed for response. | Chronic | Varchar2(85) | ▼ | | |
| | | | | | | **Antigen Presenting Cells** | This sub section stores the name of antigen presenting cells and its origin | | | | | |
| | | | | 203 | | Tissue or Cell Type | Name of the tissue or cell type of the antigen presenting cells | PBMC / Spleen / etc | Varchar2(85) | ▼ | | |
| | | | | 204 | | Origin | Origin of the cells (ex vivo, in vivo, in vitro, cell lines, clones) | ex vivo / Cell line / clone / etc | Varchar2(85) | ▼ | | |
| | | | | | | **Antigen Presentation Context** | This sub section indicates the type of process whereby a cell expresses antigen on its surface in a form capable of being recognized by T lymphocyte. | | | | | |
| | | | | 205 | | Antigen is naturally processed | This is a Boolean field that indicates whether antigen is naturally processed | Yes / No | Varchar2(1) | | | |
| | | | | | | **Antigen** | Substance that is used to detect the immune response elicited by the immunization, and from which the epitope presented by the MHC molecule, is derived. | | | | | |
| | | | 206 | | | Antigen Type | This field indicates if Antigen is same as one of the following - epitope, source protein of the epitope, source species of the epitope, or peptide containing the epitope. If antigen is none of these choices, other antigen fields that are listed below are filled out. | Epitope / Source Protein / Source Species / Peptide containing Epitope | Varchar2(85) | ▼ | | |
| | | | 207 | | | Antigen Name | Name of antigen as mentioned in the reference | NP 218-226 | Varchar2(200) | | | |
| | | | 208 | | | Gene Name | Gene name coding for the antigen used in the assay | NP | Varchar2(200) | | | |
| | | | 209 | | | Chemical Type | Categorization of antigen compound as protein, lipid, carbohydrate, DNA, RNA, organic, inorganic, etc. | Peptide, Lipid | Varchar2(35) | ▼ | | |
| | | | 210 | | | Source Species | Source Species that produced the antigen used in the assay, selected from a hierarchical list of species from NCBI taxonomy database | Influenza A Virus | Varchar2(150) | ▼ | | |
| | | | 211 | | | Species Strain | Strain of the species either as specified in NCBI Taxonomy database or recorded by the curator. | | Varchar2(85) | | | |
| | | | 212 | | | SMILES Structure | SMILES notation for 2-D structure. SMILES stand for Simplified Molecular Input Line Entry Specification. SMILES is widely used as a general-purpose chemical nomenclature and data exchange format | | Varchar2(85) | | | |
| | | | 213 | | | Sequence | Linear sequence of antigen amino acids. Other chemical structures will be captured using SMILES structure. | AYERMCNIL | Varchar2(500) | | | |
| | | | 214 | | | GenBank ID | Unique identifier of antigen used in the assay in GenBank database | K02883 | Varchar2(35) | | | |
| | | | 215 | | | Swiss Prot ID | Unique identifier of sequence of antigen used in the assay in Swiss-Prot database | P01892 | Varchar2(35) | | | |

| | | | | | | | | | | | | |
|---|---|---|---|---|---|---|---|---|---|---|---|---|
| | | | 216 | | | PDB ID | Unique PDB identifier of molecular chain representing antigen used in the assay | 1AKJ | Varchar2(35) | | | |
| | | | | | | **Carrier** | Carrier is a molecular structure that is not normally associated with the epitope. The epitope is covalently linked in vitro or in vivo, thereby in general modulating its immunogenicity, antigenicity or processing. A vector is an entity (virus, bacteria) in which the epitope is incorporated for the purpose of delivering to the cells or organism. | | | | | |
| | | | | 217 | | Carrier Name | Name of carrier as reported in the reference. | MUC1 Glycoprotein B cell Epitope | Varchar2(200) | | | |
| | | | | 218 | | Chemical Type | Categorization of carrier compound as protein, lipid, carbohydrate, DNA, RNA, organic, inorganic, etc. | Peptide | Varchar2(35) | ▼ | | |
| | | | | 219 | | SMILES Structure | SMILES notation for 2-D structure. SMILES stand for Simplified Molecular Input Line Entry Specification. SMILES is widely used as a general-purpose chemical nomenclature and data exchange format | | Varchar2(3500 ) | | | |
| | | | | 220 | | Source Species | Source species that produces the carrier, selected from a hierarchical list of species from NCBI taxonomy database and ICTV | Human | Varchar2(150) | ▼ | | |
| | | | | 221 | | Species Strain | Strain of the species either as specified in NCBI Taxonomy database or recorded by the curator. | | Varchar2(85) | | | |
| | | | | 222 | | Sequence | Linear sequence of carrier amino acids. Other chemical structures will be captured using SMILES structure. | YKQGGFLGL | Varchar2(500) | | | |
| | | | | 223 | | GenBank ID | Unique identifier of carrier in GenBank database | X91302 | Varchar2(35) | | | |
| | | | | 224 | | Swiss Prot ID | Unique identifier of carrier sequence in SWISS-PROT database | Q68840 | Varchar2(35) | | | |
| | | | | 225 | | PDB ID | Unique PDB identifier of molecular chain representing carrier | 1CWX | Varchar2(35) | | | |
| | | 226 | | | | **Special Culture Conditions** | Free text capturing any special culture conditions used in the assay | antigen presenting cells pretreated with INF-Gamma | Varchar2(500) | | | |
| | | | | | | **Assay Information** | This section captures information about the experiment used to detect the T Cell response. | | | | | |
| | | | 227 | | | Assay Type | Name of the experimental setup used to measure the immune response | ELISPOT / ICS / Cytotoxicity / etc | Varchar2(35) | ▼ | | |
| | | | 228 | | | Response Measured | Type of response measured using the assay. | INF-Gamma / Chromium Release / etc | Varchar2(100) | ▼ | | |
| | | | 229 | | | Number of Subjects Tested | Captures number of subjects that were tested | 5 | Number | | | |
| | | | 230 | | | Number of Subjects Responded | Indicates the responded subjects from the number of the subjects that were tested | 3 | Number | | | |
| | | | 231 | | | Qualitative Measurement | Qualitative assessment of the value measured using the assay as reported in the reference. | Positive / Negative | Varchar2(35) | ▼ | | |
| | | | 232 | | | Measurement Inequality | Inequality of the quantitative measurement captured. By default, measurement inequality is "=". | > / < / >= / <= / = | Varchar2(5) | ▼ | | |
| | | | 233 | | | Quantitative Measurement | Actual numerical value measured using the assay as reported in the reference. | Positive / Negative / Unknown | Number | | | |
| | | | 234 | | | Units | Standard quantities of measurement which are specific to a type of measurement. | SFC / % SL / OD / etc | Varchar2(15) | ▼ | | |
| | | | 235 | | | Location of Data | Free text mentioning the location of Assay related data in the reference. | Figure 7 / Table 2 | Varchar2(35) | | | |

| | | | | | | | | | | | | |
|---|---|---|---|---|---|---|---|---|---|---|---|---|
| | | | | | | **Epitope-MHC-TCR Complex** | This section captures all related structure fields if structure information for Epitope-MHC-TCR complex is available | | | | | |
| | | | 236 | | | Complex PDB ID | Unique four-letter PDB identifier of molecular structure representing epitope-MHC-TCR complex | 1ABC | Varchar2(35) | | | |
| | | | 237 | | | Epitope-MHC residues interacting TCR | List of epitope residues interacting with TCR | H 103, E 120 | Varchar2(200) | | | |
| | | | 238 | | | TCR residues interacting Epitope-MHC | List of TCR residues interacting with epitope-MHC | F 111, S 222 | Varchar2(200) | | | |
| | | | 239 | | | Contact area for Epitope-MHC | Contact area of epitope-MHC interacting with TCR | 800 | Number | | | |
| | | | 240 | | | Contact area for TCR | Contact area of TCR interacting with epitope-MHC | 760 | Number | | | |
| | | | 241 | | | Interacting atom pairs | Pairs of atoms involved in the interaction between epitope and TCR | A 103 OG1, B 111 N3 | Varchar2(2000) | | | |
| | | | 242 | | | Allosteric effect | This Boolean field indicates whether an allosteric effect has taken place. Allosteric effect is of or involving a change in the shape and activity of a molecule's structure that results from molecular binding with a regulatory substance at a site other than the active one where changes take place. | No / Yes | Varchar2(1) | | | |
| | | | 243 | | | Cofactor/Effector | The name of cofactor changing the epitope-TCR interaction activity if such a change has place. | Human Membrane cofactor protein | Varchar2(200) | | | |
| | | 244 | | | | Comments | Comments entered by Curator related to Assay | | Varchar2(2000) | | | |

| **Data Field #** | | | | | | **B-CELL RESPONSE** | **B Cell Response captures all the antibody responses and it's divided into two broad sub sections – Immunization and Assay.** | **Sample Values** | **Data Type** | **Drop down** | **Data Availa bility** | **Comments** |
|---|---|---|---|---|---|---|---|---|---|---|---|---|
| | | | | | | **IMMUNIZATION** | Immunization describes how the immune system is exposed to an immunogen. This sub section has categories that capture information about the immunized species, immunogen and in vivo immunization. | | | | | |
| | | | | | | **Immunized Species** | Information about the source species that is being immunized | | | | | |
| | | | 245 | | | Species | Species that received immunization by immunogen, selected from a hierarchical list of species from NCBI taxonomy database | Mouse | Varchar2(150) | ▼ | | |
| | | | 246 | | | Strain / Ethnicity | Genetic variant or an inbred line of a higher organism. The values for strain are either as specified in NCBI Taxonomy database or recorded by the curator. In case of human this field store information relating to the ethnicity individuals studied, conforming to HLA workshop conventions, if applicable. Ethnicity relates to large groups of people classed according to common racial, national, tribal, religious, linguistic, or cultural origin or background. | C57BL/6 and BALB/c | Varchar2(85) | | | |

| | | | | | | | | | | | | |
|---|---|---|---|---|---|---|---|---|---|---|---|---|
| | | | 247 | | | Sex | Sex of the individuals tested | Male | Varchar2(10) | ▼ | | |
| | | | 248 | | | Age | Age of the individuals tested | 6-15 wk age | Varchar2(35) | | | |
| | | | 249 | | | Disease Name | Disease state of the individual(s) as a result of immunization or the disease the individual(s) are associated with. IEDB conforms to International Classification of Diseases (ICD-10), if available | HCV infection | Varchar2(85) | ▼ | | |
| | | | 250 | | | Disease Stage | Disease stage of the individual(s) at the stage when effector cells are assayed for response. | Chronic | Varchar2(85) | ▼ | | |
| | | | 251 | | | Immunization Category | This field captures how the species was immunized. | Natural Infection, Inoculation, etc | Varchar2(85) | ▼ | | |
| | | | | | | **Immunogen** | Substance that is capable of inducing an immune response (antibody response or cell mediated immunity) and contains the epitope. | | | | | |
| | | | 252 | | | Immunogen Type | This field indicates if immunogen is same as one of the following - epitope, source protein of the epitope, source species of the epitope, or peptide containing the epitope. If immunogen is none of these choices, other imunogen fields that are listed below are filled out. | Epitope / Source Protein / Source Species / Peptide containing Epitope | Varchar2(85) | ▼ | | |
| | | | 253 | | | Immunogen Name | Name of immunogen as reported in the reference. | HBsAg | Varchar2(200) | | | |
| | | | 254 | | | Chemical Type | Categorization of immunogen compound as protein, lipid, carbohydrate, DNA, RNA, organic, inorganic, etc. | Peptide | Varchar2(35) | ▼ | | |
| | | | 255 | | | SMILES Structure | SMILES notation for 2-D structure. SMILES stand for Simplified Molecular Input Line Entry Specification. SMILES is widely used as a general-purpose chemical nomenclature and data exchange format | | Varchar2(3500 ) | | | |
| | | | 256 | | | Source Species | Source species that produces the immunogen, selected from a hierarchical list of species from NCBI taxonomy database and ICTV | HBV | Varchar2(150) | ▼ | | |
| | | | 257 | | | Species Strain | Strain of the species either as specified in NCBI Taxonomy database or recorded by the curator. | | Varchar2(85) | | | |
| | | | 258 | | | Sequence | Linear sequence of immunogen amino acids. Other chemical structures will be captured using SMILES structure. | | Varchar2(4000) | | | |
| | | | 259 | | | GenBank ID | Unique identifier of immunogen in GenBank database | X91302 | Varchar2(35) | | | |
| | | | 260 | | | Swiss Prot ID | Unique identifier of immunogen sequence in SWISS-PROT database | Q68840 | Varchar2(35) | | | |
| | | | 261 | | | PDB ID | Unique PDB identifier of molecular chain representing immunogen | 1CWX | Varchar2(35) | | | |
| | | | | | | **Carrier** | Carrier is a molecular structure that is not normally associated with the epitope. The epitope is covalently linked in vitro or in vivo, thereby in general modulating its immunogenicity, antigenicity or processing. A vector is an entity (virus, bacteria) in which the epitope is incorporated for the purpose of delivering to the cells or organism. | | | | | |
| | | | | 262 | | Carrier Name | Name of carrier as reported in the reference. | MUC1 Glycoprotein B cell Epitope | Varchar2(200) | | | |
| | | | | 263 | | Chemical Type | Categorization of carrier compound as protein, lipid, carbohydrate, DNA, RNA, organic, inorganic, etc. | Peptide | Varchar2(35) | ▼ | | |
| | | | | 264 | | SMILES Structure | SMILES notation for 2-D structure. SMILES stand for Simplified Molecular Input Line Entry Specification. SMILES is widely used as a general-purpose chemical nomenclature and data exchange format | | Varchar2(3500 ) | | | |
| | | | | 265 | | Source Species | Source species that produces the carrier, selected from a hierarchical list of species from NCBI taxonomy database and ICTV | Human | Varchar2(150) | ▼ | | |
| | | | | 266 | | Species Strain | Strain of the species either as specified in NCBI Taxonomy database or recorded by the curator. | | Varchar2(85) | | | |
| | | | | 267 | | Sequence | Linear sequence of carrier amino acids. Other chemical structures will be captured using SMILES structure. | YKQGGFLGL | Varchar2(4000) | | | |
| | | | | 268 | | GenBank ID | Unique identifier of carrier in GenBank database | X91302 | Varchar2(35) | | | |

| | | | | | | | | | | | | |
|---|---|---|---|---|---|---|---|---|---|---|---|---|
| | | | | 269 | | Swiss Prot ID | Unique identifier of carrier sequence in SWISS-PROT database | Q68840 | Varchar2(35) | | | |
| | | | | 270 | | PDB ID | Unique PDB identifier of molecular chain representing carrier | 1CWX | Varchar2(35) | | | |
| | | | | | | **In-Vivo Immunization** | This sub section captures fields related to immunization that is administered on living organism. | | | | | |
| | | | | | | **Formulation** | The mixture of chemicals and/or biological substances that are not covalently linked with the immunogen. | | | | | |
| | | | | 271 | | Formulation | Physical form of the mixture containing the immunogen | Liquid / Powder / Oil / Aerosol / Other | Varchar2(35) | | | |
| | | | | 272 | | Adjuvant(s) | Adjuvants are substances that are administered with the immunogen to enhance the immune response and are not covalently linked (opposed to a carrier) to the immunogen. | incomplete Freund's adjuvant | Varchar2(400) | ▼ | | |
| | | | | | | **Administration** | Process of immunizing the source species | | | | | |
| | | | | 273 | | Route | Route of administration | Direct Addition / Intravenous / Intraperitoneal / Subcutaneous / etc | Varchar2(35) | ▼ | | |
| | | | | 274 | | Dose Schedule | Captures the administration schedule including number of immunization. | 4 | Varchar2(250) | | | |
| | | 275 | | | | Comments | Comments entered by Curator that are related to Immunization | | Varchar2(2000) | | | |
| | | | | | | **ASSAY** | Assay is a quantitative or qualitative evaluation or test of substance used to describe and or measure biological response. This sub section captures fields related to B cell immune response assay. | | | | | |
| | | | | | | **Antibody** | This section captures the details of Antibody molecule and antigen binding information to the antibody along with Antigen-Antibody complex details, if available. | | | | | |
| | | | 276 | | | Antibody Name | Name of the Antibody as reported in the reference. | MAb 3120 | Varchar2(200) | | | |
| | | | 277 | | | Antibody Type | Type refers to either monoclonal or polyclonal antibody | Monoclonal | Varchar2(35) | ▼ | | |
| | | | 278 | | | Source Species | Source species of Antibody, selected from a hierarchical list of species from NCBI taxonomy database | Mouse | Varchar2(150) | ▼ | | |
| | | | 279 | | | Strain / Ethnicity | Genetic variant or an inbred line of a higher organism. The values for strain are either as specified in NCBI Taxonomy database or recorded by the curator. In case of human this field store information relating to the ethnicity individuals studied, conforming to HLA workshop conventions, if applicable. Ethnicity relates to large groups of people classed according to common racial, national, tribal, religious, linguistic, or cultural origin or background. | | Varchar2(85) | | | |
| | | | 280 | | | Immunoglobulin Domain | Fragment of Antibody that is being studied | Fab / Fc / Fd, etc | Varchar2(85) | ▼ | | |
| | | | | | | **Chain #1** | Each antibody is distinguished by certain effector functions and structural features including a unique heavy chain isotype associated with a light chain isotype. This sub section refers to heavy chain or in rare cases, a second light chain. | | | | | |
| | | | | 281 | | Isotype | Name of Isotype. Isotype describes the class, subclass, chain type and subtype of an immunoglobulin. | IgG | Varchar2(35) | ▼ | | |
| | | | | 282 | | Gen Bank ID | GenBank ID of Isotype Chain. It’s the unique identifier of gene sequence in GenBank database | AJ416332 | Varchar2(35) | | | |
| | | | | 283 | | Swiss Prot ID | Swiss-Prot ID of Isotype Chain. It’s the unique identifier of protein sequence in SWISS-PROT database | Q8VDC9 | Varchar2(35) | | | |

| | | | | | | | | | | | | |
|---|---|---|---|---|---|---|---|---|---|---|---|---|
| | | | | 284 | | PDB ID | Unique PDB identifier of molecular chain representing antibody | 1E4W | Varchar2(35) | | | |
| | | | | | | **Chain #2** | This sub section refers to light chain of antibody or in rare cases, a second heavy chain | | | | | |
| | | | | 285 | | Isotype | Name of Isotype. Isotype describes the class, subclass, chain type and subtype of an immunoglobulin. | IgG2a-kappa | Varchar2(35) | ▼ | | |
| | | | | 286 | | Gen Bank ID | GenBank ID of Isotype Chain. It's the unique identifier of gene sequence in GenBank database | AJ416332 | Varchar2(35) | | | |
| | | | | 287 | | Swiss Prot ID | Swiss-Prot ID of Isotype Chain. It's the unique identifier of protein sequence in SWISS-PROT database | Q8VDC9 | Varchar2(35) | | | |
| | | | | 288 | | PDB ID | Unique PDB identifier of molecular chain representing antibody | 1CFN | Varchar2(35) | | | |
| | | | | | | **Antigen** | Antigen is the substance that is used to detect the immune response elicited by the immunization. | | | | | |
| | | | 289 | | | Antigen Type | This field indicates if Antigen is same as one of the following - epitope, source protein of the epitope, source species of the epitope, or peptide containing the epitope. If antigen is none of these choices, other antigen fields that are listed below are filled out. | Epitope / Source Protein / Source Species / Peptide containing Epitope | Varchar2(85) | ▼ | | |
| | | | 290 | | | Antigen Name | Name of antigen used to perform the assay | ROPV L2.1 | Varchar2(200) | | | |
| | | | 291 | | | Gene Name | Gene name coding for the antigen used in the assay | NP | Varchar2(200) | | | |
| | | | 292 | | | Chemical Type | Categorization of antigen compound as protein, lipid, carbohydrate, DNA, RNA, organic, inorganic, etc. | Peptide | Varchar2(35) | ▼ | | |
| | | | 293 | | | Source Species | Source Species that produced the antigen used in the assay, selected from a hierarchical list of species from NCBI taxonomy database | Papillomavirus | Varchar2(150) | ▼ | | |
| | | | 294 | | | Species Strain | Strain of the species either as specified in NCBI Taxonomy database or recorded by the curator. | | Varchar2(85) | | | |
| | | | 295 | | | SMILES Structure | SMILES notation for 2-D structure of epitope. SMILES stand for Simplified Molecular Input Line Entry Specification. SMILES is widely used as a general-purpose chemical nomenclature and data exchange format | | Varchar2(85) | | | |
| | | | 296 | | | Sequence | Linear sequence of antigen amino acids. Other chemical structures will be captured using SMILES structure. | VGPLEVIPEAVDPAG SSIV | Varchar2(500) | | | |
| | | | 297 | | | GenBank ID | Unique identifier of antigen used in the assay in GenBank database | AF227240 | Varchar2(35) | | | |
| | | | 298 | | | Swiss Prot ID | Unique identifier of sequence of antigen used in the assay in Swiss-Prot database | Q9J028 | Varchar2(35) | | | |
| | | | 299 | | | PDB ID | Unique PDB identifier of molecular chain representing antigen used in the assay | 1JJ4 | Varchar2(35) | | | |
| | | | | | | **Carrier** | Carrier is a molecular structure that is not normally associated with the epitope. The epitope is covalently linked in vitro or in vivo, thereby in general modulating its immunogenicity, antigenicity or processing. A vector is an entity (virus, bacteria) in which the epitope is incorporated for the purpose of delivering to the cells or organism. | | | | | |
| | | | | 300 | | Carrier Name | Name of carrier as reported in the reference. | MUC1 Glycoprotein B cell Epitope | Varchar2(200) | | | |
| | | | | 301 | | Chemical Type | Categorization of carrier compound as protein, lipid, carbohydrate, DNA, RNA, organic, inorganic, etc. | Peptide | Varchar2(35) | ▼ | | |
| | | | | 302 | | SMILES Structure | SMILES notation for 2-D structure. SMILES stand for Simplified Molecular Input Line Entry Specification. SMILES is widely used as a general-purpose chemical nomenclature and data exchange format | | Varchar2(3500 ) | | | |
| | | | | 303 | | Source Species | Source species that produces the carrier, selected from a hierarchical list of species from NCBI taxonomy database and ICTV | Human | Varchar2(150) | ▼ | | |

| | | | | | | | | | | | | |
|---|---|---|---|---|---|---|---|---|---|---|---|---|
| | | | | 304 | | Species Strain | Strain of the species either as specified in NCBI Taxonomy database or recorded by the curator. | | Varchar2(85) | | | |
| | | | | 305 | | Sequence | Linear sequence of carrier amino acids. Other chemical structures will be captured using SMILES structure. | YKQGGFLGL | Varchar2(4000) | | | |
| | | | | 306 | | GenBank ID | Unique identifier of carrier in GenBank database | X91302 | Varchar2(35) | | | |
| | | | | 307 | | Swiss Prot ID | Unique identifier of carrier sequence in SWISS-PROT database | Q68840 | Varchar2(35) | | | |
| | | | | 308 | | PDB ID | Unique PDB identifier of molecular chain representing carrier | 1CWX | Varchar2(35) | | | |
| | | 309 | | | | **Special Culture Conditions** | Free text capturing any special culture conditions used in the assay | | Varchar2(500) | | | |
| | | | | | | **Assay Information** | This section captures information about the experiment used to measure the antibody response. | | | | | |
| | | | 310 | | | Materials Assayed | Materials that is being assayed in the experiment. Examples of materials assayed are serum, monoclonal antibody, purified immunoglobulin, etc | Serum | Varchar2(600) | ▼ | | |
| | | | 311 | | | Assay Type | Name of the experimental setup used to measure the immune response. | ELISA | Varchar2(35) | ▼ | | |
| | | | 312 | | | Response Measured | Type of response measured using the assay. | Antibody Production | Varchar2(100) | ▼ | | |
| | | | 313 | | | Number of Subjects Tested | Captures number of subjects that were tested | 5 | Number | | | |
| | | | 314 | | | Number of Subjects Responded | Indicates the responded subjects from the number of the subjects that were tested | 3 | Number | | | |
| | | | 315 | | | Qualitative Measurement | Qualitative assessment of the value measured using the assay as reported in the reference. | Positive / Negative | Varchar2(35) | ▼ | | |
| | | | 316 | | | Measurement Inequality | Inequality of the quantitative measurement captured. By default, measurement inequality is "=". | > / < / >= / <= / = | Varchar2(5) | ▼ | | |
| | | | 317 | | | Quantitative Measurement | Actual numerical value measured using the assay as reported in the reference. | 0.4353 | Number | | | |
| | | | 318 | | | Units | Standard quantities of measurement which are specific to a type of measurement. | Titre | Varchar2(15) | ▼ | | |
| | | | 319 | | | Location of Data | Free text mentioning the location of Assay related data in the reference. | Figure 2 / Table 1 | Varchar2(35) | | | |
| | | | | | | **Antigen-Antibody Complex** | This sub section captures all related structure fields if structure information for Antigen-Antibody complex is available | | | | | |
| | | | 320 | | | Complex PDB ID | Unique four-letter PDB identifier of molecular structure representing antigen-antibody complex | 1ABC | Varchar2(35) | | | |
| | | | 321 | | | Antigen residues interacting with antibody | List of antigen residues interacting with antibody | H 103, E 120 | Varchar2(200) | | | |
| | | | 322 | | | Antibody residues interacting with antigen | List of antibody residues interacting with antigen specified for complementarity-determining regions (CDRs) | F 111, S 222 | Varchar2(200) | | | |

| | | | | | | | | | | | | |
|---|---|---|---|---|---|---|---|---|---|---|---|---|
| | | | 323 | | | Contact area for Antigen | Contact area of antigen interacting with antibody | 800 | Number | | | |
| | | | 324 | | | Contact area for Antibody | Contact area of antibody interacting with antigen | 760 | Number | | | |
| | | | 325 | | | Interacting atom pairs | Pairs of atoms involved in the interaction between antigen and antibody | A 103 OG1, B 111 N3 | Varchar2(2000) | | | |
| | | | 326 | | | Allosteric effect | This Boolean field indicates whether an allosteric effect has taken place. Allosteric effect is of or involving a change in the shape and activity of a molecule's structure that results from molecular binding with a regulatory substance at a site other than the active one where changes take place. | No / Yes | Varchar2(1) | | | |
| | | | 327 | | | Cofactor/effector | The name of cofactor changing the antigen-antibody interaction activity if such a change has taken place. | Human Membrane cofactor protein | Varchar2(200) | | | |
| | | 328 | | | | Comments | Comments entered by Curator related to Assay | | Varchar2(2000) | | | |
